# Efficient axonal transport of endolysosomes relies on the balanced ratio of microtubule tyrosination and detyrosination

**DOI:** 10.1242/jcs.261737

**Published:** 2024-04-30

**Authors:** Anja Konietzny, Yuhao Han, Yannes Popp, Bas van Bommel, Aditi Sharma, Philippe Delagrange, Nicolas Arbez, Marie-Jo Moutin, Leticia Peris, Marina Mikhaylova

**Affiliations:** ^1^ RG Optobiology, Institute of Biology, Humboldt Universität zu Berlin, Berlin 10115, Germany; ^2^ Guest Group ‘Neuronal Protein Transport’, Center for Molecular Neurobiology, ZMNH, University Medical Center Hamburg-Eppendorf, Hamburg 20251, Germany; ^3^ Institute of Industrial Science, The University of Tokyo, Tokyo 153-8505, Japan; ^4^ Centre for Structural Systems Biology, Hamburg 22607, Germany; ^5^ Structural Cell Biology of Viruses, Leibniz Institute of Virology (LIV), Hamburg 20251, Germany; ^6^ Charité – Universitätsmedizin Berlin, Einstein Center for Neurosciences Berlin, 10117 Berlin, Germany; ^7^ Institute for Chemistry and Biochemistry, Freie Universität Berlin, Berlin 14195, Germany; ^8^ University Grenoble Alpes, Inserm U1216, CNRS, Grenoble Institut Neurosciences, 38000 Grenoble, France; ^9^ Institut de Recherche Servier, Croissy 78290, France

**Keywords:** Microtubule post-translational modification, Axonal trafficking, Axon initial segment, Tubulin-tyrosine ligase, Vasohibins

## Abstract

In neurons, the microtubule (MT) cytoskeleton forms the basis for long-distance protein transport from the cell body into and out of dendrites and axons. To maintain neuronal polarity, the axon initial segment (AIS) serves as a physical barrier, separating the axon from the somatodendritic compartment and acting as a filter for axonal cargo. Selective trafficking is further instructed by axonal enrichment of MT post-translational modifications, which affect MT dynamics and the activity of motor proteins. Here, we compared two knockout mouse lines lacking the respective enzymes for MT tyrosination and detyrosination, and found that both knockouts led to a shortening of the AIS. Neurons from both lines also showed an increased immobile fraction of endolysosomes present in the axon, whereas mobile organelles displayed shortened run distances in the retrograde direction. Overall, our results highlight the importance of maintaining the balance of tyrosinated and detyrosinated MTs for proper AIS length and axonal transport processes.

## Teaser

**Highlighted Article:** Despite opposite effects on microtubule dynamics, shifting the balance of microtubule tyrosination and detyrosination in either direction results in surprisingly similar defects in axonal organelle transport.

## INTRODUCTION

**Fig. 1.**
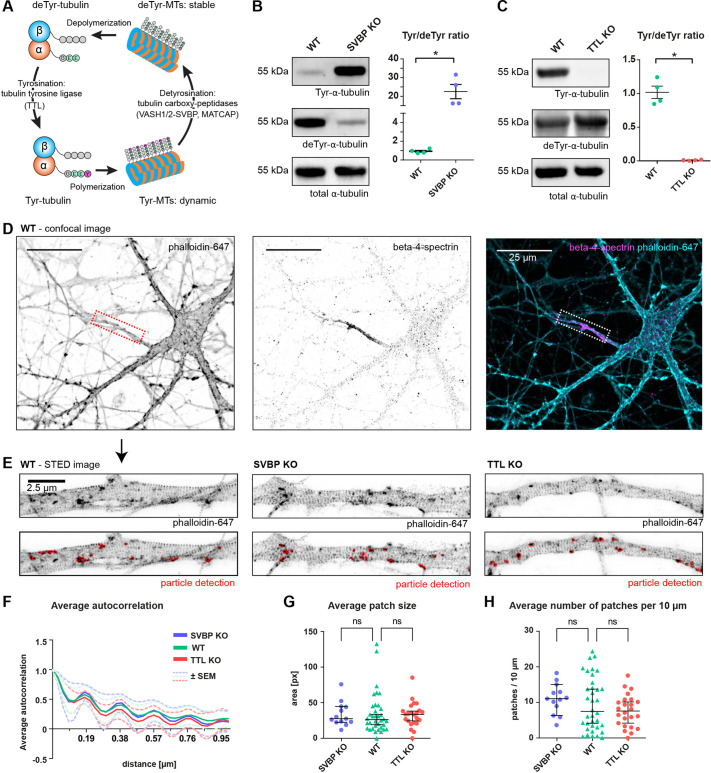
**Imbalanced MT tyrosination and detyrosination does not affect the nanostructure of the F-actin cytoskeleton in the AIS.** (A) Illustration of the tubulin tyrosination–detyrosination cycle. (B,C) Immunoblot quantifications of the ratio of tyrosinated versus detyrosinated tubulin from crude lysates of cultured mouse cortical neurons at DIV15 with SVBP-KO (B) and TTL-KO (C) compared to WT neurons. *n*=4, with three independent cultures for both WT and SVBP-KO, and four and three independent cultures for WT and TTL-KO, respectively. *P<0.05 (Mann–Whitney test with Dunn's multiple comparison test). (D) Confocal images of DIV12 neurons stained with phalloidin-647 (an F-actin marker) and with an antibody against β4-spectrin to identify the AIS (dashed box). Scale bars: 25 µm. (E) Representative STED images of F-actin in the AIS of WT, SVBP-KO and TTL-KO neurons. Lower panels show particle detection in FIJI used for quantification of F-actin patches. Scale bar: 2.5 µm. (F) Average autocorrelation of the F-actin signal periodicity in the different genotypes shows a 190 nm periodicity in WT, SVBP-KO and TTL-KO AIS. Shown is the mean±s.e.m. Calculation of autocorrelation was performed as described previously (Vassilopoulos et al., 2019, see Materials and Methods). (G) Average size (px, pixels) of F-actin patches present inside the AIS. Medians are SVBP-KO 27.65 px, WT 26.22 px, TTL-KO px 33.8. Pixel size, 30×30 nm. (H) Average number of F-actin patches per 10 µm inside the AIS. Medians are SVBP-KO 11.1, WT 7.5 and TTL-KO 7.6. For F–H, *n*=13, 36, 23 AIS for SVBP-KO (three independent cultures), WT (six independent cultures) and TTL-KO (three independent cultures). ns, non-significant (Mann–Whitney test). Error bars in B and C are mean±s.e.m.; error bars in G and H are median±95% c.i.

The complex, polarized morphology of neurons with their extensively branched neurites presents various challenges for the maintenance of cellular homeostasis. Neurons contain an elaborate intracellular recycling system within the soma, dendrites and axon that balances the transport of newly synthesized cellular components with the removal of aged and damaged ones. At the same time, transport processes need to be highly selective to maintain the molecular identities of the somatodendritic and the axonal compartments. The master regulator of polarized trafficking is a complex structure spanning the first 20–60 µm of the axon, called the axon initial segment (AIS), which selectively restricts cargo transport and is essential for the generation of action potentials (Nakada et al., 2003; Petersen et al., 2014). The AIS is composed of various elements, including transmembrane receptors, ion channels, a specialized membrane-associated periodic skeleton (MPS) rich in F-actin and spectrin, parallel microtubules (MTs) that are bundled and interlinked through tripartite-motif-containing 46 (TRIM46; van Beuningen et al., 2015), as well as dynamic EB3 (also known as MAPRE3)-positive MTs. Those components are coordinated by the master scaffolding protein ankyrin-G (AnkG, also known as ANK3; Leterrier et al., 2015). Throughout the entire cell, MT tracks provide the basis for long-distance transport, including that of recycling organelles. The clearance of damaged cellular components is mediated to a large part by acidic vesicular organelles, such as late endosomes and lysosomes, as well as a range of autophagic vesicles, which are present along the axon. Over the past years, disturbances in clearance pathways have been identified as a common denominator in many neurodegenerative diseases (Menzies et al., 2017; Pitcairn et al., 2019; Root et al., 2021; Wang et al., 2018), with axonal damage emerging as a major driver of neurodegeneration. In healthy axons, subpopulations of lysosomes delivered from the cell body ensure the timely clearance of damaged proteins (Farfel-Becker et al., 2019), and participate in signaling and RNA trafficking (Liao et al., 2019; Napolitano et al., 2022). Such MT-based transport into the axon is driven by motor proteins whose activity is regulated by selective MT post-translational modifications (PTMs; Park and Roll-Mecak, 2018). Abundant MT PTMs in neurons are tubulin tyrosination and detyrosination. Tyrosinated MT are generated from *de novo* synthesized α-tubulin, which carries the C-terminal tyrosine residue, or by tyrosine ligation to detyrosinated tubulin by the tubulin-tyrosine ligase (TTL; Fig. 1A; Ersfeld et al., 1993). MT tyrosination has been identified as a marker for dynamic microtubules (Moutin et al., 2021; Tas et al., 2017). MT detyrosination, which is largely catalyzed by the tubulin-detyrosinase complex comprising vasohibin 1 or vasohibin 2 with small vasohibin-binding protein (VASH–SVBP), occurs predominantly in the axon, where it marks a long-lived and stable MT pool (Aillaud et al., 2017; Moutin et al., 2021). Plus-end-directed kinesin motors (Cai et al., 2009; Dunn et al., 2008; Gumy et al., 2013; Homma et al., 2000; Konishi and Setou, 2009; Peris et al., 2009), as well as minus-end-directed dynein motor complexes (McKenney et al., 2016; Nirschl et al., 2016; Peris et al., 2006), are known to be sensitive to the tyrosination and detyrosination (hereafter denoted Tyr/deTyr) state of MTs, suggesting a role of MT tyrosination in the regulation of axonal molecular transport.

Here, we used two previously established knockout (KO) mouse models, TTL-KO and SVBP-KO, to characterize the influence of the microtubule Tyr/deTyr status on the structure of the AIS as well as on the axonal transport of acidic endolysosomal organelles. We found that TTL- and SVBP-KO both resulted in an overall shortening of the AIS, without affecting the F-actin nanostructure of the MPS. Furthermore, axons of both TTL- and SVBP-KO neurons contained a higher fraction of immobile endolysosomes, with the mobile fraction displaying markedly decreased run lengths in the retrograde direction. Thus, although VASH–SVBP and TTL activities are known to have opposite effects on MT dynamics (Sanyal et al., 2023), we found that the loss of either enzyme affects AIS structure and axonal trafficking in a similar way, indicating that the regulation of axonal cargo transport relies on maintaining a fine balance between MT tyrosination and detyrosination.

## RESULTS

### Alterations of the microtubule Tyr/deTyr status do not affect the AIS F-actin organization

We first quantified the ratio of tyrosinated to detyrosinated tubulin (Tyr/deTyr ratio) via immunoblot analysis of tyrosinated, detyrosinated and total α-tubulin in primary wild-type (WT), SVBP-KO and TTL-KO cultured cortical neurons obtained from mouse embryos, whose genotypes were determined via PCR (Fig. S1). As expected, the Tyr/deTyr ratio was increased in SVBP-KO neurons (22.540±3.804) and dramatically reduced in TTL-KO neurons (0.010±0.003; mean±s.e.m.; Fig. 1B,C). It has been previously shown that neurons of TTL-KO mice exhibit premature axon differentiation and formation of supernumerary axons, suggesting that enrichment of stable, detyrosinated MTs induces axon formation (Erck et al., 2005; Peris et al., 2009). In contrast, SVBP-KO neurons, devoid of VASH-SVBP carboxypeptidases, exhibit a delay in their axon development and reduced axonal length (Aillaud et al., 2017; Pagnamenta et al., 2019). To investigate whether this also has deleterious effects on the AIS structure, we conducted stimulated emission-depletion (STED) microscopy in primary hippocampal neurons to visualize the F-actin-spectrin MPS (Leterrier et al., 2015). AIS were identified by β4-spectrin immunostaining, and the nanostructure of F-actin inside the AIS was visualized using a phalloidin dye (Fig. 1D,E). We found that neither an increase (SVBP-KO) nor a reduction (TTL-KO) of the Tyr/deTyr ratio interfered with the formation of the MPS or the presence of F-actin patches in the AIS (Fig. 1E). F-actin and β4-spectrin form an intercalated submembrane scaffold complex with a 190 nm periodicity (Vassilopoulos et al., 2019), which was equally present in WT, SVBP-KO and TTL-KO neurons, as judged by an autocorrelation analysis of the longitudinal F-actin signal along the AIS (Fig. 1F). Given that the cargo barrier function of the AIS relies specifically on F-actin patches present inside the AIS (Song et al., 2009; Watanabe et al., 2012), we compared average patch size and number of patches per 10 µm between the different genotypes, and found that none of those metrics was significantly different from WT in the two KOs (Fig. 1G,H). We therefore consider it unlikely that the F-actin-dependent barrier function of the AIS is impaired in either SVBP- or TTL-KO.

### SVBP-KO and TTL-KO both lead to a shortening of the AIS

**Fig. 2.**
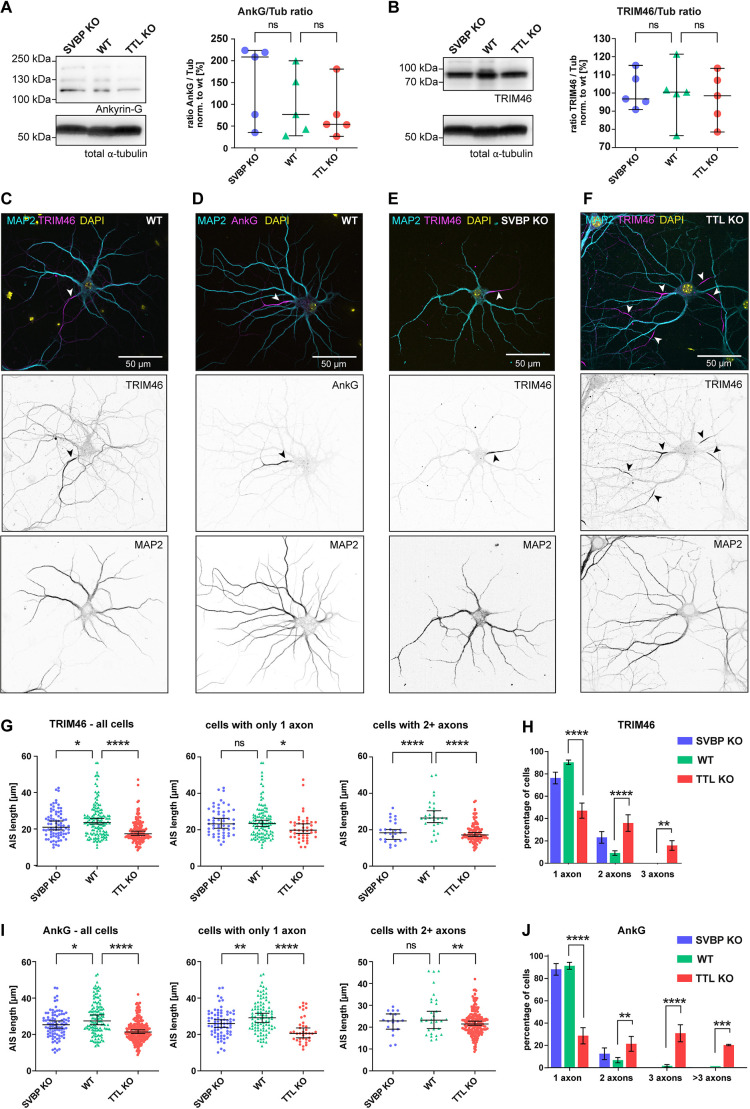
**Both SVBP-KO and TTL-KO lead to shorter AIS in hippocampal neurons.** (A) Western blot analysis of AnkG levels in SVBP-KO, TTL-KO and WT DIV11 primary cortical neurons. Left, representative western blot against AnkG and total α-tubulin (Tub) as loading control. Right, quantification for the ratios of AnkG to tubulin (AnkG/Tub) normalized to the mean of WT. (B) Western blot analysis of TRIM46 levels in SVBP-KO, TTL-KO and WT DIV11 primary cortical neurons. Left, representative western blot against TRIM46 and total α-tubulin as loading control. Right, quantification for ratios of TRIM46 to tubulin (TRIM46/Tub) normalized to the mean of WT. For A and B, individual values and median with 95% c.i. are shown, *n*=5 independent cultures, ns, not significant (Kruskal–Wallis test with Dunn's multiple comparisons test). (C,D) Example images of WT DIV12 mouse hippocampal neurons stained for the AIS markers TRIM46 (B) or AnkG (C) together with MAP2 (dendritic marker), and DNA (DAPI). Arrowheads indicate the AIS. (E,F) Representative images of DIV12 TTL-KO (D) and SVBP-KO (E) neurons stained for MAP2, TRIM46 and DAPI. Arrowheads indicate the AIS. (G,I) AIS length of TRIM46-stained (G) and AnkG-stained (I) neurons from SVBP-KO, WT, and TTL-KO mice. Individual values and median with 95% c.i. are shown. Left panel, graphs show all cells analyzed together. Middle and right panels, graphs show cells with only 1 axon and cells with 2+ axons analyzed separately. (H,J) Quantification of neurons with supernumerary axons in the different genotypes as judged by TRIM46 (H) and AnkG (J) staining. Error bars indicate mean±s.e.m. In G–J, TRIM46, *n*=68, 93 and 123 AIS for SVBP-KO (two independent cultures), TTL-KO (five independent cultures) and WT (six independent cultures); AnkG, *n*=84, 110 and 108 AIS for SVBP-KO (three independent cultures), TTL-KO (four independent cultures) and WT (five independent cultures). ns, not significant; *P<0.05; **P<0.005; ***P<0.0005; ****P<0.0001 [Kruskal–Wallis test with Dunn's multiple comparisons test (G,I); two-way repeated measures ANOVA with Dunnett's multiple comparisons test (H,J)].

Apart from the cargo filtering function of the AIS, polarized trafficking is additionally instructed by uniformly oriented axonal MTs, which are tightly bundled by the protein TRIM46 inside the AIS (van Beuningen et al., 2015). We therefore decided to compare the distribution of two AIS markers, AnkG and TRIM46, in proximal axons of KO and WT mouse hippocampal neurons. To determine whether expression levels of either protein were altered in KO neurons, we quantified the AnkG to tubulin (Fig. 2A) and the TRIM46 to tubulin (Fig. 2B) ratios via immunoblot analysis and found no significant difference between WT and KOs (Fig. 2A,B). Next, to investigate whether formation of the AIS was impaired in SVBP- or TTL-KO, we quantified AnkG and TRIM46 signals in immunostainings using a MATLAB script based on previously established criteria (Grubb and Burrone, 2010; Fig. S2). Representative images of WT and KO neurons stained for TRIM46 (and AnkG for WT) are presented in Fig. 2C–F. In TTL-KO neurons, both markers indicated a significantly shortened AIS (AnkG, 21.4 µm; TRIM46, 17.9 µm) compared to WT (AnkG: 27.5 µm, TRIM46: 24.0 µm; Fig. 2G,I, left). The SVBP-KO showed a similar effect on AnkG (25.4 µm; Fig. 2I, left), and a non-significant trend towards a shorter AIS with TRIM46 (22.9 µm; Fig. 2G, left). In line with previous observations (Erck et al., 2005), the absence of TTL resulted in a strong supernumerary axon phenotype – more than 50% of the analyzed neurons had two or more AIS emerging from soma or dendrites (Fig. 2F), while the SVBP-KO produced no such effect (Fig. 2H,J). The formation of supernumerary axons was not a determining factor in the reduction of average AIS length, given that it occurred both in cells with only one axon and cells with supernumerary axons (Fig. 2G,I, middle and right panels).

### Lysotracker-positive organelles in the distal axon are proteolytically active

**Fig. 3.**
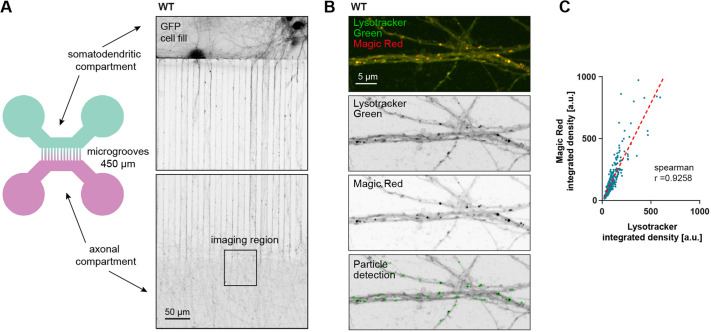
**Lysotracker-positive vesicles in distal axons are degradatively active.** (A) Illustration of the two-compartment MFC used for live-imaging of axonal trafficking (left panel), and microscopy image of DIV12 mouse hippocampal neurons expressing GFP (right panel). (B) Confocal image of axons inside MFC, labeled with Lysotracker Green and Magic Red. (C) Integrated density values of Lysotracker correlate with the integrated density of Magic Red; 815 particles were analyzed in three separate locations of one independent neuron culture in an area of ∼0.2 mm². The dotted red line indicates the calculated linear regression (*y*=1578×*x*+4156); Spearman's correlation coefficient, *r*=0.9258.

Next, we set out to analyze how shifting the balance in the MT Tyr/deTyr ratio would impact axonal cargo transport. We decided to investigate the trafficking of lysosomal organelles because they play an important role in axonal homeostasis (Farfel-Becker et al., 2020), and lysosomal transport has been shown to be affected by the Tyr/deTyr state of MTs (Mohan et al., 2019). For this, we used two-compartment microfluidic chambers (MFCs; Fig. 3A) and Lysotracker, a pH-sensitive dye that non-discriminately labels acidic organelles, including late endosomes, lysosomes and autophagic vesicles. As there is some debate in the literature regarding bona fide degradation-competent lysosomes being present in distal axonal compartments (Farfel-Becker et al., 2019; Lie et al., 2021), we measured the correlation between Lysotracker fluorescence and fluorescence of Magic Red – a specific substrate of the lysosomal, proteolytically active form of cathepsinB (Ni et al., 2022), which fluoresces upon proteolytic cleavage (Fig. 3B). We found that the fluorescence intensity of Lysotracker correlated strongly with the fluorescence intensity of Magic Red (R^2^=0.9258; Fig. 3B,C), indicating that most Lysotracker-positive vesicles in the distal axon contained active cathepsinB. However, given that both Magic Red and Lysotracker label a range of acidic endolysosomal compartments, it should be noted that a spectrum of degradative organelles will be visualized with these probes. For simplicity, from this point on we will use the term ‘endolysosomes’ to refer to these Lysotracker-positive organelles, which is also found in the wider literature (Keren-Kaplan et al., 2022; Roney et al., 2022; van Bommel et al., 2019).

### Microtubule tyrosination status affects axonal retrograde trafficking of endolysosomes

**Fig. 4.**
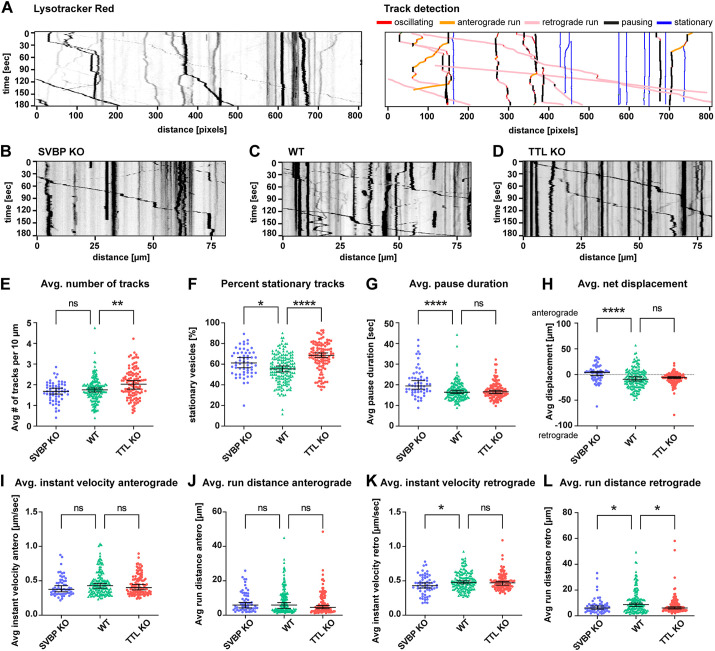
**The retrograde trafficking efficiency of endolysosomes is similarly decreased in the distal axons of TTL**-**KO and SVBP-KO hippocampal neurons.** (A) Left, representative kymograph from individual Lysotracker-treated axons; each trace represents a single organelle. Right, kymograph analysis presented on the left using a Python script (see Materials and Methods for details). (B–D) Representative example kymographs for SVBP-KO (B), WT (C) and TTL-KO neurons (D). See also Movies 1–3. (E–L) Summary of vesicle trafficking properties during the imaging period. (E) Average number of all detected vesicles per 10 µm (mobile and stationary). (F) Percentage of stationary vesicles. (G) Average pause duration. (H) Average net displacement in anterograde or retrograde direction. (I,J) Average anterograde instant velocities and run distances. (K,L) Average retrograde instant velocities and run distances. For E–L, individual values and median with 95% c.i. are shown. *n*=58, 164 and 114 axons from SVBP (three independent experiments), WT (six independent experiments) and TTL-KO (five independent experiments), respectively. ns, not significant; *P<0.05; **P<0.005; ****P<0.0001 (Kruskal–Wallis test with Dunn's multiple comparisons test).

We next investigated whether the Tyr/deTyr status of MTs had a differential impact on endolysosomal organelle transport in the axonal compartment of hippocampal neurons grown in two-compartment MFCs. To minimize investigator bias and inconsistencies of manual analysis, we developed a semi-automated analysis workflow to analyze kymographs from individual axons (Fig. 4A; Fig. S3; https://github.com/HU-Berlin-Optobiology/AIS-project). Using this setup, we compared various trafficking parameters between SVBP-KO (Fig. 4B; Movie 1), WT (Fig. 4C; Movie 2) and TTL-KO (Fig. 4D; Movie 3). The investigated parameters included the number of mobile and immobile vesicles, pausing times, net direction of transport, velocity, and run lengths (Fig. 4E–L). In TTL-KO neurons, a significantly higher median number of endolysosomes was detected compared to WT (2.0 and 1.8, respectively; Fig. 4E). We observed a strong increase in the proportion of completely stationary organelles in both SVBP (61%) and TTL-KO (69%) compared to WT (56%; Fig. 4F). Endolysosomes in SVBP-KO neurons (19.5 s) took longer pauses than WT (16.4 s), which was not the case for TTL-KO (16.7 s; Fig. 4G). In the axon, anterograde transport is carried out by plus-end-directed kinesin motors, whereas retrograde movement is mediated by minus-end-directed dynein motors. We analyzed the net displacement in the anterograde versus retrograde direction and observed that in WT, endolysosomes underwent a net retrograde displacement (median −9.1 µm). This was also true for TTL-KO (median −5.9 µm), but interestingly not for SVBP-KO, which showed a net anterograde displacement (median +3.9 µm; Fig. 4H). Anterograde transport, including median velocity (WT 0.43 µm/s; Fig. 4I) and run distances (WT 5.88 µm; Fig. 4J), was unaffected by lack of either TTL or SVBP. Retrograde transport showed reduced median velocities in SVBP-KO (0.43 µm/s) compared to WT (0.48 µm/s; Fig. 4K), and significantly shorter median run lengths in both genotypes (SVBP-KO 6.11 µm; TTL-KO 6.14 µm, WT 8.71 µm; Fig. 4L). Taken together, our findings indicate that specifically retrograde trafficking of endolysosomes is influenced by the Tyr/deTyr state of microtubules.

### shRNA knockdown of TTL leads to an increase in stationary Lysotracker-positive vesicles

**Fig. 5.**
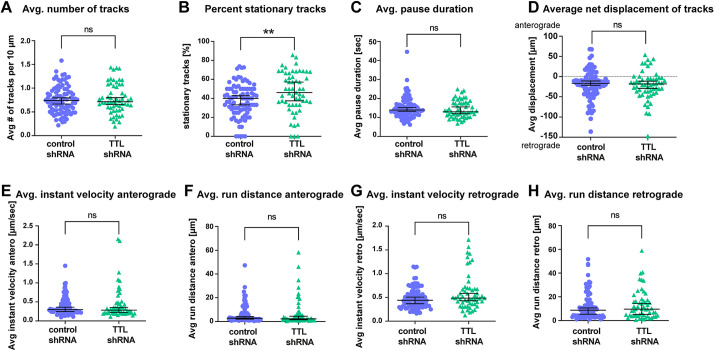
**Acute shRNA knockdown of TTL leads to an increase in stationary endolysosomes.** Quantification of (A) average number of all detected vesicles per 10 µm (mobile and stationary), (B) percentage of stationary vesicles, (C) average pause duration, (D) percentage net displacement in anterograde or retrograde direction, (E,F) average anterograde instant velocities and run distances, and (G,H) average retrograde instant velocities and run distances for control and TTL shRNA knockdown neurons. Individual values and median with 95% CI are shown. *n*=90 and 57 axons from control (three independent cultures) and TTL shRNA (three independent cultures). ns, not significant; **P<0.005 (Mann–Whitney test).

Given that defects in early neuronal polarization observed in TTL-KO neurons (Fig. 2F,H,J) could potentially impact axonal trafficking at later stages, we wanted to test whether an increase in the relative amount of detyrosinated MTs after the establishment of neuron polarity would lead to a similar trafficking phenotype. We therefore conducted trafficking experiments where we knocked down the TTL enzyme after 5 days *in vitro* (DIV5) in WT mouse hippocampal neurons, i.e. after axon and dendrite specification (Dotti et al., 1988). Expression of the shRNA knockdown construct reduced TTL levels in DIV12–13 neurons by 20% (Fig. S4A) and led to a significant decrease in the Tyr/deTyr tubulin ratio (to 85% of control) in transfected cells (Fig. S4B). Using this approach on neurons grown in MFCs, we conducted the same Lysotracker-trafficking experiments as described above, as summarized in Fig. 5. Most of the investigated parameters were similarly unaffected by TTL knockdown as by TTL-KO, such as pause duration (Fig. 5C), net displacement (Fig. 5D), anterograde and retrograde velocities (Fig. 5E,G) and anterograde run distance (Fig. 5F). Contrary to what was seen in TTL-KO, TTL shRNA knockdown did not affect the average numbers of Lysotracker-labeled vesicles (Fig. 5A) or retrograde run distances (Fig. 5H). However, following TTL shRNA knockdown, the relative amounts of stationary vesicles were significantly increased (median 46%, control 40%; Fig. 5B), similar to what was seen in TTL-KO (median 69% versus 56% in WT; Fig. 4F). This indicates that even a moderate perturbation in the Tyr/deTyr MT balance caused by TTL knockdown disrupts the efficient trafficking of axonal endolysosomes.

## DISCUSSION

In this study, we aimed to investigate whether MT tyrosination and detyrosination play an instructive role in polarized axonal transport, specifically focusing on the AIS structure and long-range secretory organelle trafficking. We compared neurons from two knockout mouse lines – one lacking tubulin tyrosine ligase, leading to a strong increase in the Tyr/deTyr ratio, and the other lacking SVBP, resulting in a substantial decrease of the Tyr/deTyr ratio. Confocal and STED imaging revealed that both knockouts resulted in a shortened AIS, without affecting the F-actin organization. Subsequently, using microfluidic devices, we compared axonal trafficking in the different genotypes, using acidic endolysosomes as a representative example of highly motile organelles abundant in the axon and crucial for axonal function. Despite opposite effects on microtubule dynamics, manipulating the balance of MT tyrosination to detyrosination in either direction resulted in surprisingly similar defects in axonal organelle transport. A less drastic reduction in TTL expression through shRNA knockdown partially recapitulated the TTL-KO phenotype, leading to an increase of completely immobile organelles. This suggests that even a moderate shift in the Tyr/deTyr MT ratio affects the trafficking of axonal endolysosomes.

Previous studies using TTL-KO and SVBP-KO mice have mostly focused on morphological and behavioral phenotypes. Notably, the KO of TTL causes perinatal death, which is in part due to a disorganization of neuronal networks. Although general organogenesis was normal in these mice, they show breathing defects and ataxia, likely caused by perturbed neocortical layering and cortico-thalamic loop formation (Erck et al., 2005). Conditional TTL-KO in the forebrain leads to viable and fertile mice that have deficits in spatial learning and increased anxiety. These mice show several CNS defects, such as incomplete development of the corpus callosum and the anterior commissures, and, in the hippocampus, a reduced number of dendritic spines and impaired synaptic plasticity (Hosseini et al., 2022). In contrast to TTL-KO, SVBP-KO mice are viable and fertile, but their brain volume is decreased overall by 7% with over 30% reduction in some white matter tracts. They present behavioral defects like mild hyperactivity, lower anxiety and impaired social behavior, although memory defects have not been reported (Pagnamenta et al., 2019). Until now there has been no study directly addressing the electrophysiological properties of TTL and SVBP-KO neurons in terms of probability of action potential generation. In light of our results showing that both TTL-KO and SVBP-KO neurons have shorter AIS compared to WT, it would be interesting to investigate whether this also affects the threshold for action potential generation or activity-induced homeostatic plasticity of the AIS (Evans et al., 2015).

Super-resolution imaging revealed no changes in the organization of F-actin structures within the AIS. Like WT, the AIS of TTL- and SVBP-KO contained the characteristic MPS with a periodicity of ∼190 nm. Also, the size and density of F-actin patches were unaltered, which suggests that the cargo filtering function of the AIS is intact in KO neurons (Watanabe et al., 2012). Interestingly however, alterations in the MT Tyr/deTyr state had distinct effects on the AIS marker proteins TRIM46 and AnkG. Immunoblot analysis of KO cultures did not reveal any significant changes in TRIM46 or AnkG proteins levels, although levels varied quite strongly between individual cultures. When quantifying the length distribution of TRIM46 and AnkG along the AIS, we found that the apparent length of the AIS was reduced in both SVBP- and TTL-KO, with loss of TTL having a stronger effect than SVBP. Although TRIM46 and AnkG mark distinct subdomains within the AIS and control different molecular mechanisms, they are both essential for the formation of the AIS during neuronal development and function in interdependent pathways. TRIM46 is a very early marker for neuronal polarization and localizes to the future axon even before axon specification and before AnkG clustering (van Beuningen et al., 2015) through an unknown mechanism. After axon specification, even though there is no known direct link between AnkG and TRIM46, knockdown of AnkG causes redistribution of TRIM46 (van Beuningen et al., 2015). This indicates that in adult neurons, TRIM46 localization to the AIS depends on AnkG, and factors that perturb AnkG will likely also affect TRIM46. Apart from that, TRIM46 is known to be carried into the AIS by the kinesin-2 motor complex (Ichinose et al., 2019), which is sensitive to the MT Tyr/deTyr state (Gumy et al., 2013), so the observed reduction of TRIM46 recruitment to the AIS could also be mediated by a less efficient transport in the TTL-KO condition. Finally, as an MT-binding protein, TRIM46 itself might favor specific MT PTMs or tubulin isotypes that are only present at the proximal axon, even though none of the modifications known to date specifically mark the position of the AIS (Janke and Kneussel, 2010). Factors that delimit the length of the TRIM46-associated bundles inside the AIS, or the extent of the AIS itself, are currently still unknown (Yang et al., 2019), and are interesting targets for future investigations.

To examine the effect of MT Tyr/deTyr on long-distance transport processes in the distal axonal compartment of hippocampal neurons, we opted to use Lysotracker to label acidic organelles, which are abundant and highly motile in this compartment. We observed largely balanced bi-directional transport of Lysotracker-positive organelles, with a slight predominance of retrograde transport in WT and TTL-KO neurons. These results are in contrast to a previous report, which showed that in developing cortical neurons, acidified and lysosomal-associated membrane protein 1 (LAMP1)-positive organelles move almost exclusively in the retrograde direction (Lie et al., 2021). LAMP1 is a transmembrane protein that localizes to mature lysosomes, but also labels a wide range of other autophagic, endolysosomal and secretory vesicles (Cheng et al., 2018). Using a ratiometric pH indicator, Lie et al. (2021) report that highly acidic LAMP1 vesicles exhibited predominantly retrograde transport, though most were immobile, whereas weakly acidic LAMP1 vesicles traveled bidirectionally at a wide range of velocities, in both retrograde and anterograde directions (Lie et al., 2021). It is interesting to note this difference to the behavior of Lysotracker-positive vesicles in our study, which likely stems from the different ranges of organelle identities that were observed, but could also be explained by the different neuronal subtypes used in the studies (hippocampus versus cortex), as well as different ages of the cultures and culture conditions.

Intriguingly, in SVBP-KO neurons, we observed a change in overall net displacement directionality, from net retrograde displacement to net anterograde displacement. This might be a reflection of the fact that it is mainly retrograde transport that seems to be affected by changes in the MT Tyr/deTyr state in our model system – endolysosomes in both SVBP-KO and TTL-KO neurons showed decreased retrograde run distances, and additionally decreased retrograde velocity in SVBP-KO. Owing to the uniform polarity of axonal MTs, all retrograde trafficking is mediated by the dynein–dynactin complex (Cason et al., 2021; Gowrishankar et al., 2021). It is known that the dynactin subunit p150Glued (also known as DCTN1) and the dynein regulator CLIP-170 (also known as CLIP1) preferentially bind to tyrosinated MTs, which in WT neurons are enriched in the distal tip of the axon, to initiate retrograde transport (McKenney et al., 2016; Nirschl et al., 2016; Peris et al., 2006). Although an *in vitro* study has shown that the subsequent continuation of processive motility is unaffected by the Tyr/deTyr state of the MT track (McKenney et al., 2016), the interaction of p150Glued with the MT lattice promotes processive motility of the dynein complex over a state of passive diffusion (Feng et al., 2020). Given that p150Glued preferentially interacts with tyrosinated MTs, this could potentially explain the effects we see in TTL-KO. To our knowledge, no studies have quantified the processivity, binding affinity or velocity of dynein–dynactin on tyrosinated versus detyrosinated MTs. The mechanistic explanation of why we observed reduced retrograde movement speed and run length in the SVBP-KO is intriguing and remains open at this point, and it is likely to be complicated by an altered environment of microtubule-associated proteins (Monroy et al., 2020) or by a shift in abundancies of other MT PTMs. Of note, a study on lysosome transport on tyrosinated versus detyrosinated MTs in non-neuronal epithelial cells has shown that LAMP2-labeled lysosomes are preferentially recruited to detyrosinated MT by the motor protein kinesin-1, and that they show reduced motility on detyrosinated MT compared to on tyrosinated MT (Mohan et al., 2019). Although axonal LAMP1- or Lysotracker-positive lysosomes in hippocampal neurons are also transported by kinesin-1 (Farías et al., 2017), we did not observe any specific effects on anterograde (i.e. kinesin-mediated) transport in either the SVBP-KO or TTL-KO condition. This could be explained by differences in endolysosomal transport regulation between epithelial cells and neurons, and is an interesting point for further investigations in the future.

The fact that changes in the Tyr/deTyr state of MT seem to predominantly affect retrograde, but not anterograde trafficking is also interesting considering that the maturation of both endolysosomes and autophagosomes is tightly linked with their retrograde transport through the axon (Cason et al., 2021; Lie et al., 2021). It is likely that perturbances in the retrograde transport process caused by a change in the Tyr/deTyr ratio would also affect organelle maturation. In this regard, future experiments that focus on organelle identity and maturation state along the axon in combination with the MT Tyr/deTyr status would be highly informative. The defects in axonal transport that we observed in this study most likely also contribute to the altered axon development described for TTL- and SVBP-deficient neurons (Pagnamenta et al., 2019; Peris et al., 2009), and hence in the neural tract abnormalities of TTL- and SVBP-KO mice (Erck et al., 2005; Pagnamenta et al., 2019). Finally, it has been recently discovered that TTL expression is reduced in cases of both sporadic and familial Alzheimer's disease, and neurons harboring the familial APP-V717I Alzheimer mutation exhibit decreased MT dynamics (Peris et al., 2022), suggesting a potentially altered regulation of MT-based cargo transport in these diseased neurons. In this regard, TTL shRNA knockdown could be useful as a model to discriminate early developmental defects in the knockout from trafficking phenotypes caused by the reduction in MT tyrosination.

## MATERIALS AND METHODS

### Animals

All experiments involving animals were carried out in accordance with the European Communities Council Directive (2010/63/EU). Experiments using tissue from WT C57BL/6J mice were carried out in accordance with the national Animal Welfare Act of the Federal Republic of Germany (Tierschutzgesetz der Bundesrepublik Deutschland, TierSchG) approved by the local authorities of the city-state Hamburg (Behörde für Gesundheit und Verbraucherschutz, Fachbereich Veterinärwesen) and the animal care committee of the University Medical Center Hamburg-Eppendorf, as well as of the Office for Health and Social Welfare (Landesamt für Gesundheit und Soziales, LAGeSo, Berlin, Germany) and the control of the animal welfare officers of the Humboldt University of Berlin (reference number T HU-05/22). All experiments involving knockout mice were conducted in accordance with the policy of the Grenoble Institut Neurosciences (GIN) and in compliance with the French legislation. Mice homozygous for an inactivated tubulin tyrosine ligase allele (referred to as TTL-KO) and for an inactivated small vasohibin-binding protein allele (referred to as SVBP-KO), were obtained as previously described (Erck et al., 2005; Pagnamenta et al., 2019) and maintained in a C57BL/6 genetic background.

### Preparation of microfluidic chambers

PDMS and curing agent (SYLGARD 184 Silicone Elastomer) were mixed thoroughly in a 10:1 ratio in a falcon tube and centrifuged for 5 min at 4000 ***g*** to remove air bubbles. The PDMS was then filled into the epoxy molds and placed under vacuum for 30 min, then polymerized for 3–4 h at 60°C. The cured PDMS patterns were carefully removed from the molds, the wells were cut out using a 4 mm biopsy punch, and the shape of the chambers was cut using a scalpel knife. They were then placed pattern-side down on sticky tape to protect from dust and stored for up to 2 weeks. To prepare for use, the microfluidic chambers (MFCs) were removed from the sticky tape and vortexed in 96% ethanol for 3–5 min, then washed 3× with MilliQ water and placed under a sterile hood for air drying. For surface activation, the MFCs were placed in a plasma cleaner, pattern-side up, together with 28 mm glass coverslips placed into 35 mm tissue-culture dishes (without lids), and treated with plasma for 30 s. The chambers were then placed upside down on the glass coverslips as quickly as possible, then the lids were placed on the dishes and the chambers were baked for another 15–30 min at 60°C. For sterilization, the MFCs inside the culture dishes were placed in a sterile hood under UV light for 20 min. They were then placed inside a 15 cm Petri dish together with a piece of sterilized Whatman filter paper wetted with MilliQ water to prevent drying out of the chambers. For neuronal culture, the chambers were either coated with poly-L-lysine as described below (Lysotracker for kymograph analysis, Fig. 4) or with 0.01 mg/ml poly-L-ornithine-hydrobromide (Sigma, Cat# P3655) in PBS overnight at room temperature (RT), washed twice with MilliQ water and incubated with 2.8 µg/ml laminin (Bio-Techne, Cat# 3400-010-02) in PBS for 1 h at 37°C (Lysotracker and Magic Red analysis, Fig. 3). Then they were washed twice with MilliQ water and stored with Hank's balanced salt solution (HBSS, Sigma, Cat# H9269) at 37°C.

### Coating of culture dishes for hippocampal neurons

Glass coverslips and microfluidic chambers were coated with 1 mg/ml poly-L-lysine (Sigma #P-2636) in borate buffer (3.1 mg/ml boric acid, 4.75 mg/ml borax, pH 8.50) overnight at room temperature. The dishes were then subjected to a short rinse, a long rinse (1 h), and two short rinses with sterile water. Finally, DMEM (Life Technologies, 31966047) plus 10% horse serum (Life Technologies, 26050088) was added to the dishes and brought to 37°C. For STED imaging, high-precision coverslips (Marienfeld, 117580) were used.

### Preparation of knockout mouse hippocampal cultures

Embryonic day (E)18.5 mouse embryos were collected in sterile filtered 1× PBS. The brains were dissected, and the hippocampi were individually collected in 1× Hank's balanced salt solution (HBSS, Gibco #14185-045). Using a sterile plastic pipette, the two hippocampi of one embryo were taken in 2 ml of 1× HBSS. Subsequently, 200 µl of 10× trypsin (Gibco #14185-045) was added, and the solution was gently agitated and incubated at 37°C for 15 min without mixing during incubation. After incubation, the hippocampi were rinsed once with 1× HBSS at room temperature. The supernatant was removed and 500 µl of DMEM plus 10% horse serum at RT was added. Mechanical dissociation was performed by pipetting up and down using a P1000 pipette with a 200 µl pipette tip on top of the 1000 µl tip, up to a maximum of 10 times. For immunostainings, 20 µl of the 500 µl cell suspension were plated on glass coverslips in a 24-well plate containing 1 ml of DMEM plus 10% horse serum. For microfluidic chambers, 35 µl of the 500 µl cell suspension were centrifuged at 1000 ***g*** for 2 min, 30 µl of the supernatant were removed and the cells were resuspended in the remaining 5 µl, and injected into the microfluidic chamber. The four wells of the chamber were then filled up with DMEM plus 10% horse serum. After plating, the cultures were incubated at 37°C with 5% CO_2_ for 2 h, before the culture medium was replaced with MACS Neuro Medium (Miltenyi Biotec #130-093-570) supplemented with B27 (Life Technologies #17504044).

### Preparation of knockout mouse cortical cultures

Cortices from E18.5 mouse embryos were collected and treated as hippocampal neuron cultures. After trypsin incubation and mechanical dissociation, 1×10^6^ cells were seeded in 0.5 mg/ml poly-L-lysine-coated 35 mm plastic dishes with DMEM+10% horse serum for 2 h, which was then replaced with MACS Neuro Medium supplemented with B27.

### Genotyping

PCR amplifications were performed on alkaline lysates of toe clips or tail cuts of E18.5 mouse embryos. Briefly, mouse tissue was incubated for 30 min at 95°C in alkaline solution (NaOH 25 mM, EDTA 0.2 mM, pH 12.0). Neutralization was performed by adding 40 mM Tris, pH 5.0. Lysates were then analyzed by PCR with corresponding primers and Econo Taq PLUS Green Mix (Euromedex). Primers pairs for testing the TTL mouse strain were 5′-GGCGACTCCATGGAGTGGTGG-3′ and 5′-CCCAACATCACATTCTCCAAATATCAAAG-3′ (TTL WT, 1032 bp) and 5′-GATTCCCACTTTGTGGTTCTAAGTACTG-3′ and 5′-CCCAACATCACATTCTCCAAATATCAAAG-3′ (TTL KO, 900 bp). The four primers were used in a single reaction (Fig. S1A). Primers pairs for testing SVBP mouse strain were 5′-GATCCACCTGCCCGGAAA-3′ and 5′-TTTCTTCCAGCACCCTCTCC-3′ (SVBP WT, 170 bp) and 5′-TTTCTTCCAGCACCCTCTCC-3′ and 5′-CAAACCATGGATCCACGAAA-3′ (SVBP KO, 167 bp). These latter reactions were done separately (Fig. S1B). The following amplification protocols were used: TTL, 95°C for 5 min, 35 cycles of [95°C for 1 min, 50°C for 1 min, 72°C for 1 min], 72°C for 2 min; SVBP, 95°C for 5 min, 33 cycles of [95°C for 30 s , 50°C for 30 s, 72°C for 30 s], 72°C for 2 min. DNA was analyzed on 1.2% and 2% agarose gels for TTL and SVBP, respectively. Pictures of whole agarose gels are shown in Fig. S6.

### Immunoblotting

For quantification of tyrosination and detyrosination levels (Fig. 1), cortical neurons were cultured for 15 days *in vitro*, lysed in Laemmli buffer and boiled at 96°C for 5 min. The total protein contents were equilibrated using stain-free 4–15% gels (Bio-Rad) and then quickly transferred onto nitrocellulose using a Trans-Blot Turbo Transfer System (Bio-Rad). Immunoblots were developed using specific primary antibodies against detyrosinated α-tubulin (1:8000), tyrosinated α-tubulin (1:5000) and total α-tubulin (1:8000) (see Table S1). Specific fluorescent secondary antibodies conjugated to Alexa Fluor 488 or Cy5 (Jackson Laboratories) were used and analyzed with a ChemiDoc™MP Imaging System (Bio-Rad) using Image Lab software (stain-free gel and fluorescence protocol) for quantification. For each lane of the blot, the software measures the integrated intensity of the band corresponding to the antigen of interest. The signal of each modified tubulin (tyrosinated or detyrosinated) was normalized to the signal of total α-tubulin of the same lane and the ratio of normalized signals was calculated. One neuronal culture per embryo was processed as indicated and for each neuronal culture, three independent blots were performed. For analysis of AnkG and TRIM46 protein levels (Fig. 2), primary cortical neurons from SVBP-KO, TTL-KO and WT animals were lysed in Laemmli buffer at DIV11 and samples were boiled at 96°C for 5 min. The proteins were separated by SDS-PAGE on 10% gels and transferred onto PVDF membranes (BioRad Immun-Blot PVDF, Cat# 1620177). After transfer, the membranes were blocked with 5% milk powder in TBS with 0.1% Tween 20 (TBS-T) for 1 h at RT. Then, the membranes were incubated with antibodies against AnkG (EMD Millipore, Cat# MABN466, mouse, 1:500 dilution), TRIM46 (Proteintech, Cat# 30298-1-AP, rabbit, 1:2000 dilution) and α-tubulin (Sigma, Cat# T5168, mouse 1:2000 dilution) diluted in TBS with 0.0005% sodium azide (TBS-A) at 4°C overnight. Membranes were washed three times in TBS-T (each wash at RT for 10 min) and incubated with secondary antibodies anti-mouse-IgG conjugated to HRP (Dianova, Cat# 115-035-146, goat, 1:10,000 dilution) or anti-rabbit IgG conjugated to HRP (Dianova, Cat# 111-035-144, goat, 1:10,000 dilution) diluted in 5% milk powder in TBS-T at RT for 90 min. Afterwards, blots were washed twice with TBS-T and once with TBS (each wash at RT for 10 min) and developed using enhanced chemiluminescence solution made in house (100 mM Tris-HCl pH 8.8, 1.25 mM luminol, 2 mM 4-iodophenylboronic acid, 4.9 mM H_2_O_2_) (Haan and Behrmann, 2007). Signals were detected with a BioRad ChemiDoc MP Imaging System and analyzed with the analyze gel function in Fiji software. For AnkG, all three visible bands above 100 kDa were measured and summed up. The ratio of AnkG and TRIM46 integrated intensity to integrated intensity of α-tubulin (detected on the same membrane) was formed and values were normalized to the mean of WT. Spectra HR (Thermo Fisher Scientific, Cat# 26625) was used as a protein ladder. Original western blot images can be found in the Figs S5 and S6. Details on antibodies can be found in Table S1.

### WT mouse neuron culture for TTL shRNA knockdown

P0 ‘wildtype’ C57BL/6J mouse pups were decapitated by cervical dislocation, and embryo heads were cut to dissect the brains. The brains were dissected in ice-cold HBSS (Sigma, H9269), and the hippocampi of several animals were collected in 450 µl cold HBSS. 50 µl trypsin (Gibco, 12499-015) were added to a final concentration of 0.025% and incubated for 15 min at 37°C. After 2× washes with 500 µl warm HBSS, the hippocampi were resuspended in 1 ml warm plating medium [DMEM (Gibco, 41966-029) with 10% FBS and 1% penicillin-streptomycin (Invitrogen, 15140122)] and mechanically dissociated by carefully pipetting up and down through two different needles with different pore sizes (yellow 20G and brown 26G) with a 2 ml syringe, up to four times each. The cells were counted manually using a Neubauer chamber by staining 15 µl of the cell suspension with Trypan Blue. For microfluidic chambers, 70,000–80,000 cells were centrifuged at 1000 ***g*** for 2 min, the supernatant was removed, and the cells were resuspended in 5 µl plating medium, and injected into the microfluidic chamber. The four wells of the chamber were then filled up with plating medium, and the cultures were incubated at 37°C with 5% CO_2_ for 2 h before the culture medium was replaced with growth medium [Neurobasal A (Thermo Fisher, 12349015) supplemented with 1× B27 Plus (Thermo Fisher, A3582801), 4 mM Glutamax (Gibco, 35050061) and 1 mM sodium pyruvate (Gibco, 11360070)]. The cells were then transduced on DIV5 by the addition of adeno-associated virus (AAV) containing either TTL shRNA or control shRNA expression vectors (Table S1), directly into the cell medium. At 7–8 days after transduction (DIV12–13; Fig. S4), neurons were imaged with Lysotracker Red as described below.

### Dual imaging of Magic Red and Lysotracker Green

Magic Red™ (Biomol, Immunochemistry Technologies, ICT-937) was reconstituted with DMSO, aliquoted and stored at −80°C according to the manufacturer's instructions. Primary hippocampal neurons were prepped from C57BL/6 P0 mice. 80,000 cells were plated into the somatodendritic compartment of a two-compartment MFC. At DIV14 the MFC was placed in a stage-top incubator (okolab) at 37°C, 5% CO_2_ and 90% humidity atmosphere at the microscope. LysoTracker™ Green DND-26 (Thermo Fisher Scientific, Invitrogen™, L7526) and Magic Red™ were added to the cell medium of both somatodendritic and axonal compartments of the MFC to a final dilution of 1:10,000 for LysoTracker™ and 1:250 for Magic Red™. After a 2–3 min incubation, confocal imaging was performed with a Nikon Eclipse Ti-E Visitron SpinningDisk confocal microscope controlled by VisiView software (Visitron Systems). The samples were imaged using a 100× TIRF objective (Nikon, ApoTIRF 100×/1.49 oil) resulting in a pixel size of 65 nm with 488 and 561 nm excitation laser. Lasers were coupled to a CSU-X1 spinning-disk (Yokogawa) unit via a single-mode fiber. Emission light was collected through a filter wheel with filters for GFP (Chroma ET525/50 m) and RFP (ET609/54 m). *Z*-stacks were acquired with a pco.edge 4.2 bi sCMOS camera (Excelitas PCO GmbH) with 350 nm step size in 16-bit depth. For image analysis, the FIJI plugin ‘ComDet’ (v.0.5.5) was used to detect particles in the green channel (Lysotracker-positive organelles). The detected ROIs were then used to measure fluorescence intensity in both the green and red channels.

### Immunocytochemistry

Cells were fixed in 4% Roti-Histofix (Carl Roth) and 4% sucrose in PBS for 10 min at RT, and washed three times with PBS, before they were permeabilized in 0.2% Triton X-100 in PBS for 10 min. The cells were then washed three times in PBS and blocked for 45 min at RT with blocking buffer (BB; 10% horse serum, 0.1% Triton X-100 in PBS). Incubation with primary antibodies was performed in BB at 4°C overnight. After washing three times in PBS, cells were incubated with corresponding secondary antibodies (see Table S1) in BB for 1 h at RT and washed again for three times for 10 min each time in PBS. For STED imaging, the coverslips were subjected to a second overnight incubation step with 1:100 phalloidin-Atto647N (Sigma, 65906) in PBS and washed again for three times for 10 min each time in PBS. As a final step, coverslips were post-fixed in 2% Roti-Histofix and 2% sucrose in PBS for 10 min at RT, washed three times for 10 min each time with PBS, and mounted on microscope slides using Mowiol. Mowiol was prepared according to the manufacturer's instructions [9.6 g mowiol 4–88 (Carl-Roth, 0713.1), 24.0 g glycerine, 24 ml H_2_O, 48 ml 0.2 M Tris pH 8.5, 2.5 g DABCO (Sigma-Aldrich, D27802)]. Details on antibodies and other reagents can be found in Table S1.

### STED imaging and analysis

Samples were imaged using an inverted microscope (Axio observer Z1 Zeiss) with a 100×/1.46 objective. The AIS in stained cells was identified by an accumulation of the marker β4-spectrin, and the ROI for STED imaging was selected accordingly. STED was performed using the STEDYCON module driven by Imspector software (Abberior Instruments). To achieve STED super-resolution (30 nm pixel size) of STAR Orange and Alexa Fluor 594, the 561 nm laser was used at 20–30% laser power with the 775 nm depletion laser at 79.95%, and for phalloidin–Atto-647N, the 640 nm laser was used at 5–10% laser power with the 775 nm depletion laser at 46.5%. The number and size of F-actin patches in the phalloidin-Atto647N STED images were quantified using a Python script written in house for FIJI based on the particle detection plugin as described in Han et al., 2023 preprint (see ‘synpo_det_1.0’, https://github.com/HU-Berlin-Optobiology/AIS-project). Briefly, to remove background structures from phalloidin images, the AIS was manually outlined in FIJI using the polygon selection tool, and the ROIs were fed into the ‘synpo_det’ script with the following detection parameters: intensity threshold (‘pa_thre’)=0.6, size threshold (‘area_thre’)=0.7. The periodicity of the submembrane F-actin cytoskeleton was analyzed as described in Vassilopoulos et al. (2019), using the code available on GitHub (https://github.com/cleterrier/Process_Profiles/blob/master/Autocorrelation_.js ). ROIs for the script were manually drawn over a stretch of the AIS with visibly good phalloidin staining using the ‘segmented line’ tool in FIJI.

### Confocal imaging of AIS markers

Z-stack images of fixed neurons were acquired on Leica TCS SP8 and Leica TCS SP5 confocal microscopes with 488 nm, 568 nm and 633 nm excitation lasers using a 63.0×/1.40 NA oil objective. The pixel size was set to 90 nm and *Z*-steps varied between 250 and 350 nm.

### Live imaging of Lysotracker in KO and WT neurons

Live imaging of KO cultures grown in MFCs was conducted on DIV12. Lysotracker Red DND-99 (Thermo Fisher Scientific, L7528) was added to the somatodendritic compartment of the MFC to a final dilution of 1:10,000. Lysotracker-stained vesicles became visible in the axonal compartment a few minutes later. Imaging was conducted at 37°C and 5% CO_2_ on a Zeiss Axio Observer coupled to a spinning disk confocal system (CSU-W1-T3; Yokogawa) connected to an electron-multiplying CCD camera (ProEM+1024, Princeton Instruments). Images were taken every 1 s for 180 s with a 63×/1.46 NA oil immersion objective.

### Imaging of Lysotracker in TTL knockdown neurons

Live imaging of TTL shRNA knockdown cultures grown in MFCs was conducted on DIV12–13. Lysotracker Red DND-99 (Thermo Fisher Scientific, L7528) was to the somatodendritic compartment of the MFC to a final dilution of 1:10,000. Lysotracker-stained vesicles became visible in the axonal compartment a few minutes later. shRNA-transfected axons were identified via GFP expression. Imaging was conducted at 37°C and 5% CO_2_ with a spinning disc confocal microscope (Nikon ECLIPSE Ti) controlled by VisiView software (Visitron Systems) and equipped with the following components: Spinning Disk (Yokogawa), solid-state lasers (488, 561, 647 and 405 nm), an EM-CCD camera (Hamamatsu, Digital Camera C9100), and with a 100×/NA 1.45 objective. The image acquisition rate was 1 fps over 3 min.

### Semi-automated analysis of kymographs

Axons of Lysotracker-treated neurons grown in MFC were imaged as above. Timelapse image stacks were processed using FIJI/ImageJ. For shRNA-transfected neurons, individual axons were identified by their GFP expression. For knockout neurons, individual axons were identified by a brightfield image taken before live imaging. Only axons where the directionality could be established (i.e. axons directly coming out of the MFC's microgrooves) were chosen. Individual axons were manually traced using the segmented line tool with a 10-pixel width (pixel size=0.175 µm). Kymographs were then generated from those ROIs using the KymoResliceWide plugin (https://github.com/UU-cellbiology/KymoResliceWide). The kymographs were then used as input for the bidirectional KymoButler deep learning software (Jakobs et al., 2019; https://github.com/elifesciences-publications/KymoButler) run in Wolfram Mathematica with the following settings: detection threshold=0.2, decision threshold=0.5, minimum particle size=30, minimum frame number=20. Automatically detected traces from KymoButler were loaded into a MATLAB program written in-house (https://github.com/HU-Berlin-Optobiology/AIS-project) to overlay them with the original kymograph image, and manually corrected when there was a visible error. The researcher conducting the manual correction was not aware of the data identity. The corrected tracks were then exported as lists of *x-y* coordinates, and a Python-based program was used to extract various parameters from the data (see Fig. S3; https://github.com/HU-Berlin-Optobiology/AIS-project.git). In short, the program assigned ‘movement’ if the *x*-coordinate (*x*-axis=space) changed from one frame to the next (*y*-axis=time) and assigned ‘no movement’ if the *x*-coordinate stayed the same from one frame to the next. Cutoff values were then set to identify'runs’ as a continuous movement for five frames, and ‘pause’ for continuous non-movement for five frames (i.e. a ‘pause’ was defined as a period in which a particle defined as mobile, which had at least one processive run during the imaging period, did not move for five or more consecutive frames). Anything below those cutoffs was assigned as ‘oscillating’. A track that did not contain any runs was identified as completely stationary. ‘Runs’ were further divided into anterograde runs and retrograde runs. For each individual identified track, the program calculated the duration of runs and pauses, the run distance and run velocity, and exported those values both individually and as averaged values per each track. Only the run distance and velocity of periods where the particles underwent processive runs for at least five consecutive frames were taken into account. For each mobile particle, the net displacement in anterograde or retrograde direction was determined. If a particle moved both in the retrograde and anterograde direction during the imaging period, it might end up having no net displacement (‘none’) despite being mobile. The threshold for net displacement was set to 5 pixels (0.875 µm).

### Statistical analysis

All statistical analysis was conducted in GraphPad Prism 8.02, and Prism 10.2.0. Prism scatter plot appearance was set to ‘Standard’ (width of distribution of points proportionate to the number of points at that *y*-value). Error bars show the median±95% confidence interval, unless specified otherwise. Data was tested for Gaussian distribution using the D'Agostino and Pearson normality test and accordingly subjected to parametric or non-parametric statistical tests. Statistical analysis of differences between two groups was performed using unpaired two-tailed Student's *t*-tests for populations with Gaussian distribution, or Mann–Whitney's test for non-Gaussian distributions. When comparing three or more univariate samples, we used one-way ANOVA, or the non-parametric Kruskal–Wallis test. Post-hoc comparisons following Kruskal–Wallis test were done with the non-parametric Dunn test.

## Supplementary Material



10.1242/joces.261737_sup1 Supplementary information

## Data Availability

All relevant data can be found within the article and its supplementary information.
