# Supplementary material for: Efficient axonal transport of endolysosomes relies on the balanced ratio of microtubule tyrosination and detyrosination
Source: J Cell Sci. 2024 Apr 30;137(8):jcs261737. doi: 10.1242/jcs.261737 (PMC11112122; doi:10.1242/jcs.261737)
Supplement: Supplementary information [file joces-137-261737-s1.pdf]

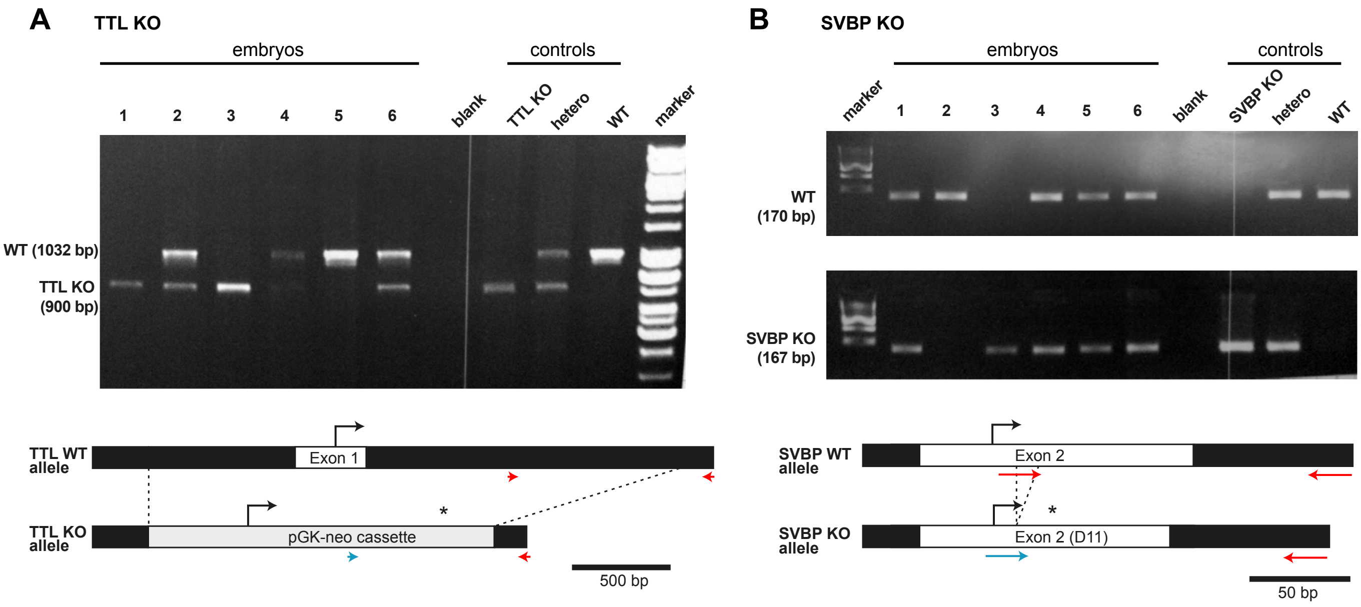


**Fig. S1. TTL-KO and SVBP-KO genotyping and protein levels of AnkG and TRIM46.** A) Exon 1 of TTL was replaced by a neomycin resistance cassette driven by phosphoglycerate kinase I promoter (pGK-neo) as described in Erck et al. (2005). B) A frameshift was introduced in exon 2 of SVBP, causing a premature stop codon (*), as described in Pagnamenta et al. (2019). For details on genotyping primers, see Material & Methods.

Journal of Cell Science • Supplementary information

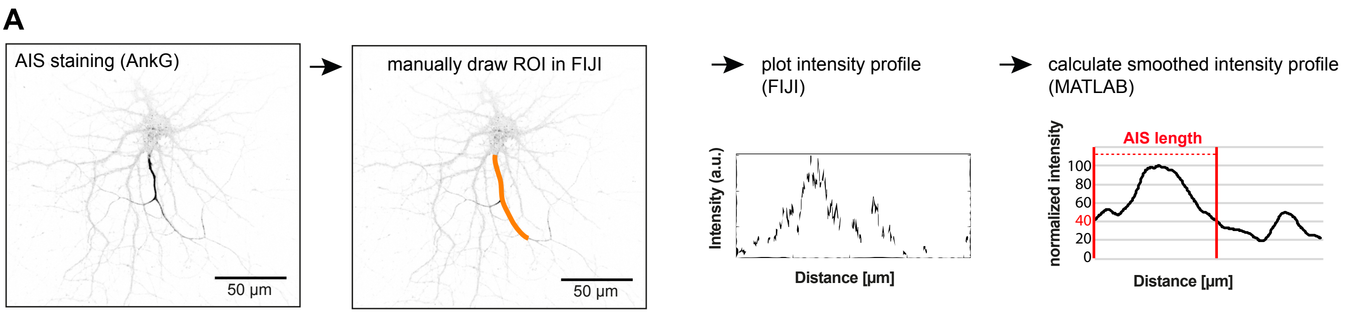


**Fig. S2. Overview of the workflow for AIS length calculation.**

A) Neurons were stained with an AIS marker (AnkG or TRIM46). A ROI was drawn manually from the cell body over the entire length of the marker region in FIJI. Raw intensity profiles from FIJI were smoothed in MATLAB using a 3-pixel sliding window and normalized to 100%. Start and end of the AIS were defined as the points where the normalized intensity crossed above or below 40% of the maximum intensity. Scale bar = 50 um.

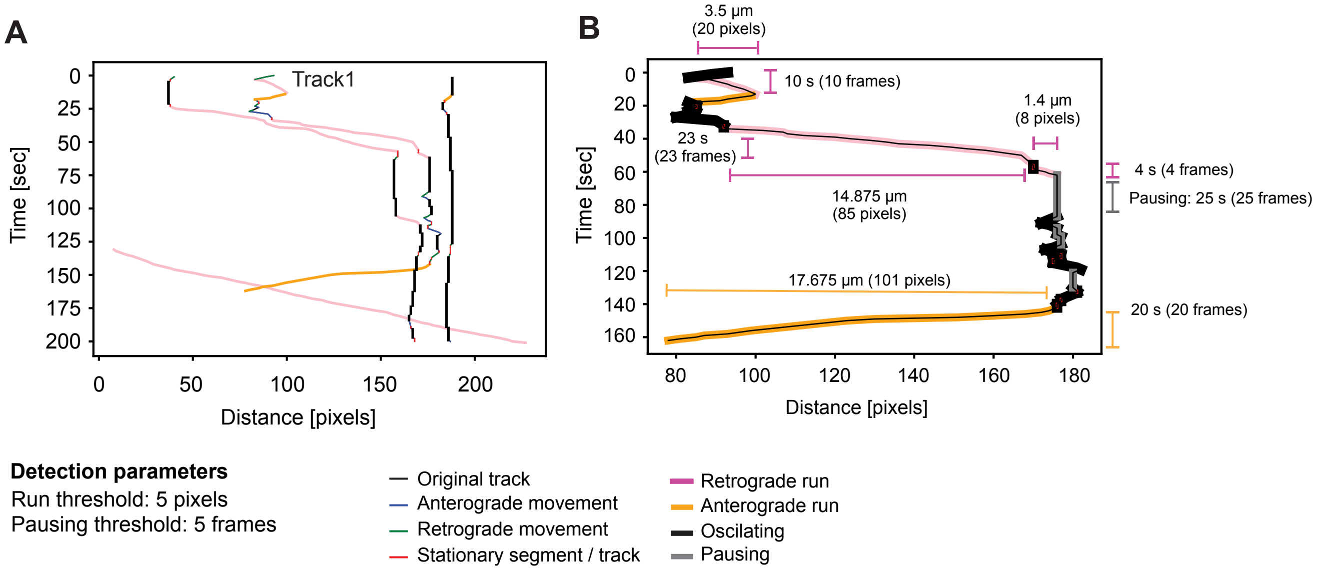


**Fig. S3. Semi-automated analysis of lysotracker-positive vesicles.** A) Example of kymograph reconstruction via the KYMOA program (https://github.com/HU-Berlin-Optobiology/AIS-project.git) showing Lysotracker-vesicle trajectories in the axonal compartment of a mouse hippocampal neuron. Each track represents the trajectory of one Lysotracker-positive vesicle. B) Annotation of track 1. In total, 3 processive retrograde runs, 2 processive anterograde runs, 3 pausing events and 6 oscillating movements were identified with the detection parameters indicated at the bottom of the figure.

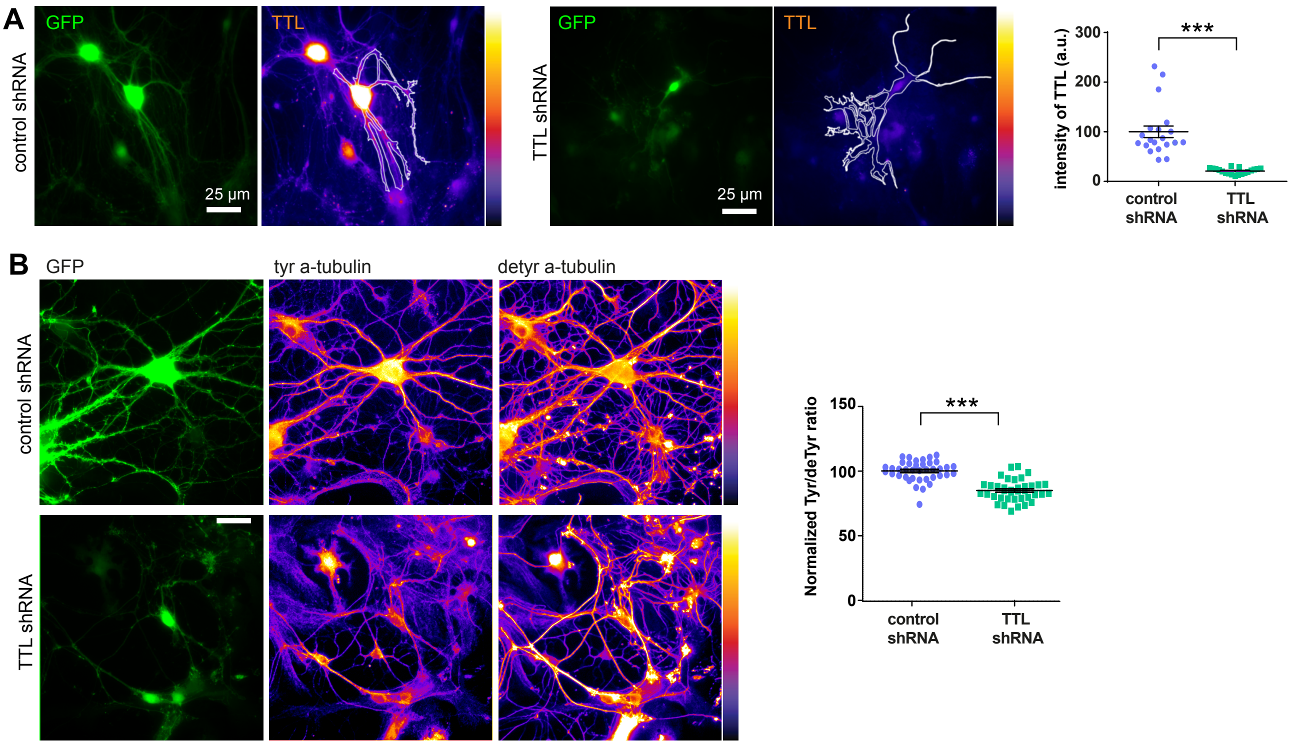


**Fig. S4. Acute shRNA knockdown of TTL in mouse hippocampal neurons. A)** Knockdown of TTL leads to reduced detected TTL levels in DIV 14 neurons. Cell outline is marked with white mask. **B)** Knockdown of TTL decreases the Tyr/deTyr ratio in DIV14 neurons. Scale bars = 25 µm. (A) n = 20, 19 cells from control (1 independent culture) and TTL shRNA (1 independent culture). (B) n = 39, 38 cells from control (2 inde-pendent cultures) and TTL shRNA (2 independent cultures). Error bars show mean ± SEM.

Journal of Cell Science • Supplementary information

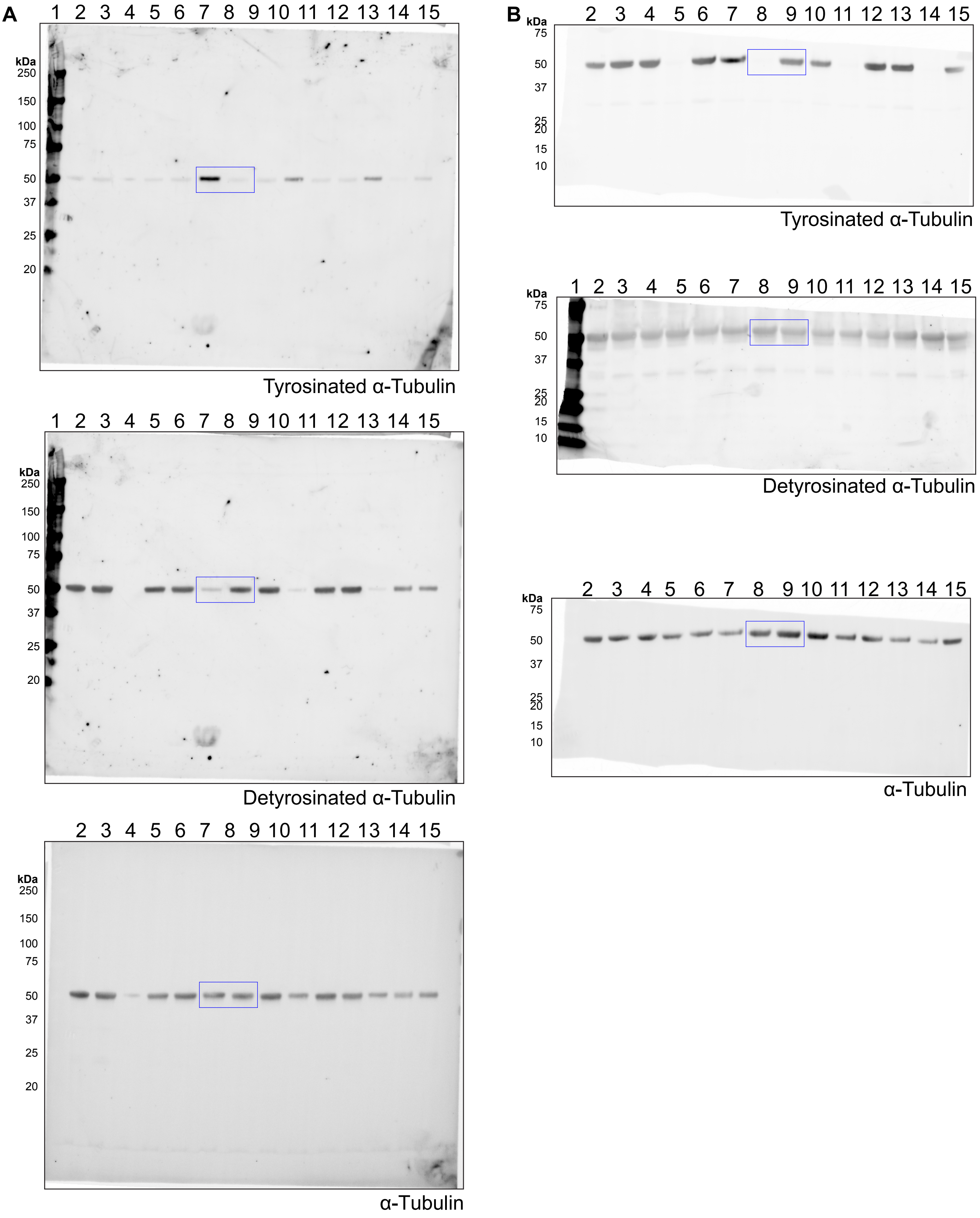
A
1 2 3 4 5 6 7 8 9 10 11 12 13 14 15
kDa
250
150
100
75
50
37
25
20
Tyrosinated α-Tubulin
Detyrosinated α-Tubulin
2 3 4 5 6 7 8 9 10 11 12 13 14 15
α-Tubulin
B
kDa
75
50
37
25
20
15
10
Tyrosinated α-Tubulin
Detyrosinated α-Tubulin
α-Tubulin

**Fig. S5. Western blot Transparency corresponding to Fig. 1.**

**A)** Whole membranes corresponding to Figure 1 B), blue boxes correspond to insets shown in Fig. S1 C), insets have been flipped horizontally and contrast was increased. 1 Protein Ladder, 2 WT, 3 SVBP het., 4 SVBP KO, 5 WT, 6 SVBP het., 7 SVBP KO, 8 WT, 9 SVBP het., 10 SVBP KO, 11 WT, 12 SVBP het., 13 SVBP KO, 14 WT, 15 WT+TTL. **B)** Whole membranes corresponding to Figure 1 C), blue boxes correspond to insets shown in supplemental Figure 1 C), insets have been flipped hori-zontally and contrast was increased. 1 Protein Ladder, 2 CI, 3 WT, 4 TTL het., 5 TTL KO, 6 WT, 7 TTL het., 8 TTL KO, 9 WT, 10 TTL het., 11 TTL KO, 12 WT, 13 TTL het., 14 TTL KO, 15 CI.

Journal of Cell Science • Supplementary information

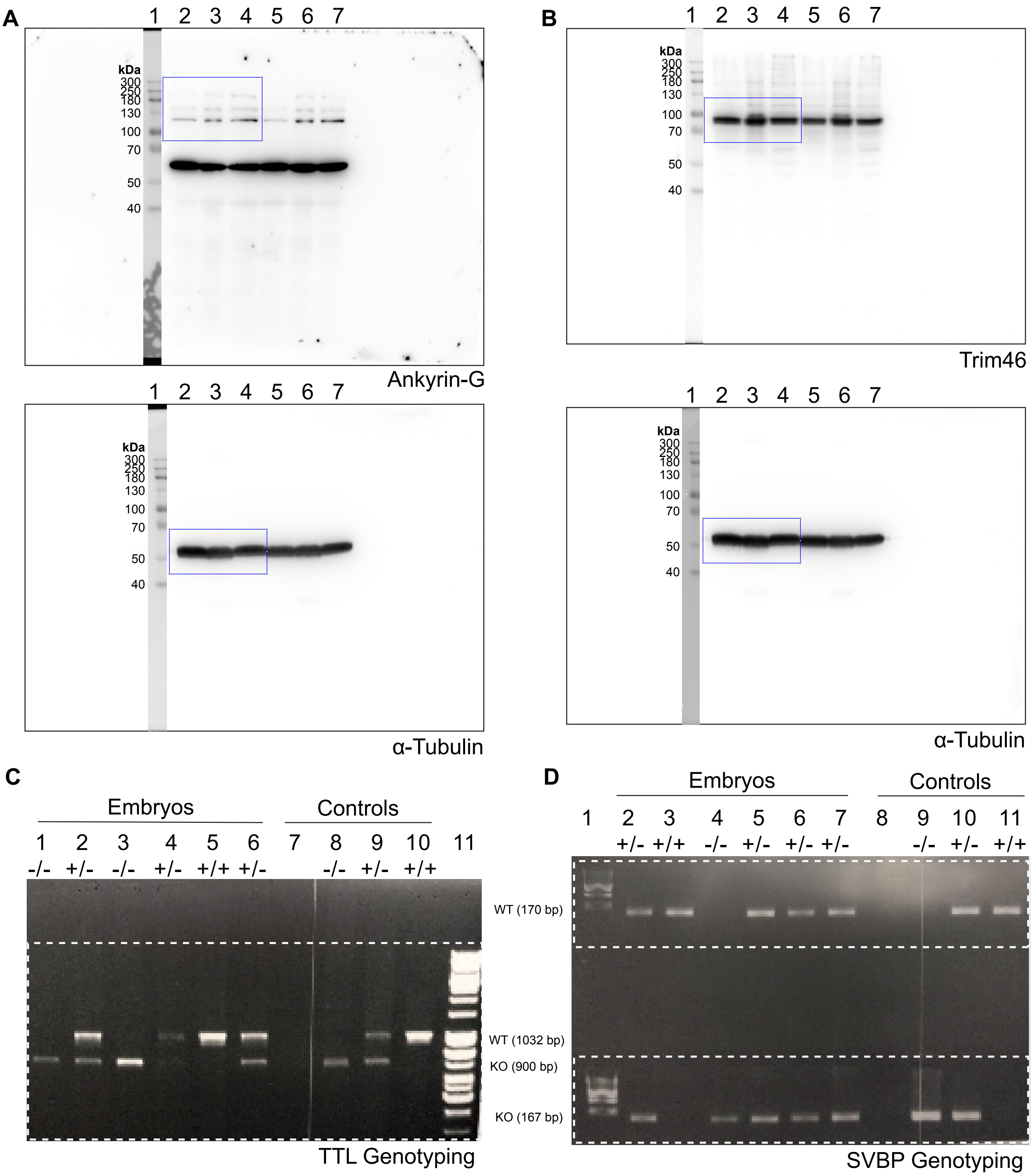


Journal of Cell Science • Supplementary information

**Fig. S6. Western blot and agarose gel transparency corresponding to Fig. 2 and Fig. S1**

**A)** Whole membranes corresponding to Figure 2 A), blue boxes correspond to insets shown in Figure 2 A), insets have been flipped horizontally. 1 Spectra High-Range, 2 TTL KO, 3 WT, 4 SVBP KO, 5 TTL KO, 6 WT, 7 SVBP KO. **B)** Whole membranes corresponding to Figure 2 B), blue boxes correspond to insets shown in Figure 2 B), insets have been flipped horizontally. 1 Spectra High-Range, 2 TTL KO, 3 WT, 4 SVBP KO, 5 TTL KO, 6 WT, 7 SVBP KO. **C)** Whole agarose gel corresponding to Supplemental Figure 1 A), dashed line box corresponds to inset shown in Supplemental Figure 1 A). 1 Embryo #1: TTL KO, 2 Embyro #2: TTL het., 3 Embryo #3: TTL KO, 4 Embyro #4: TTL het., 5 Embyro #5: WT, 6 Embryo #6: TTL het., 7 blank control, 8 TTL KO control, 9 TTL het. control, 10 WT control, 11 DNA marker. **D)** Whole aga-rose gel corresponding to Supplemental Figure 1 B), dashed line boxes correspond to insets shown in Supplemental Figure 1 B). 1 DNA marker, 2 Embyro #1: SVBP het., 3 Embryo #2: WT, 4 Embyro #3: SVBP KO, 5 Embyro #4: SVBP het., 6 Embryo #5: SVBP het., 7 Embryo #6: SVBP het., 8 blank control, 9 SVBP KO control, 10 SVBP het. control, 11 WT control.

Journal of Cell Science • Supplementary information

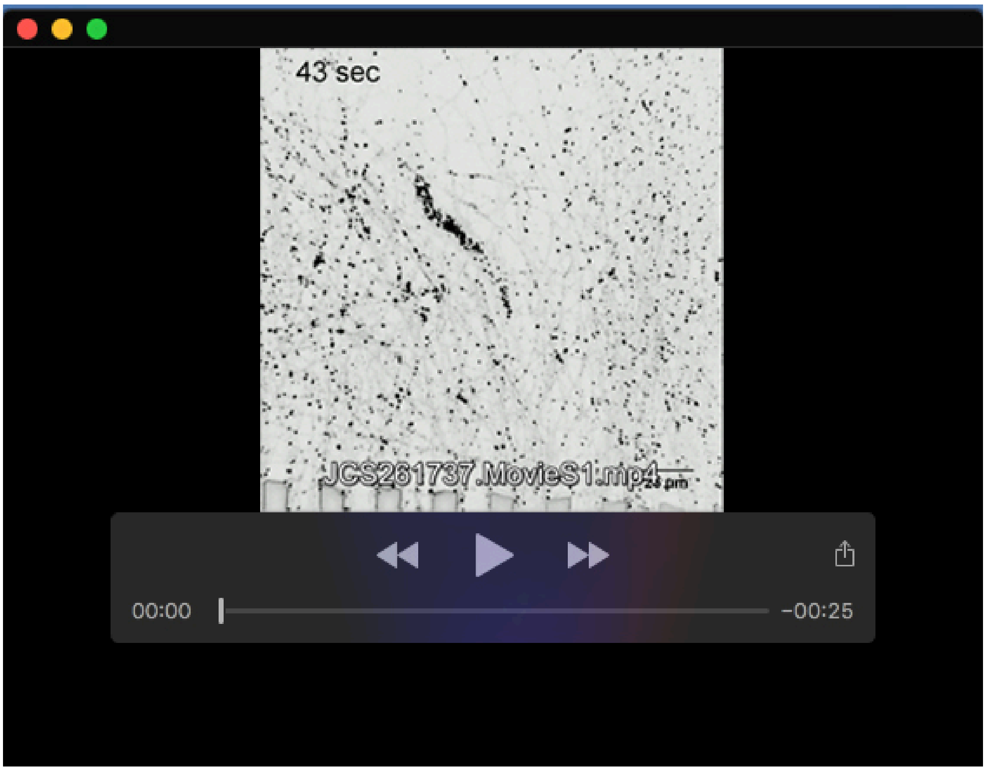


**Movie 1.** Corresponding to Fig. 4B. Movement of lysotracker-positive vesicles in the axonal compartment of SVBP KO neurons grown in 2-compartment MFCs imaged at 1 frame per second. Scale bar = 25 µm.

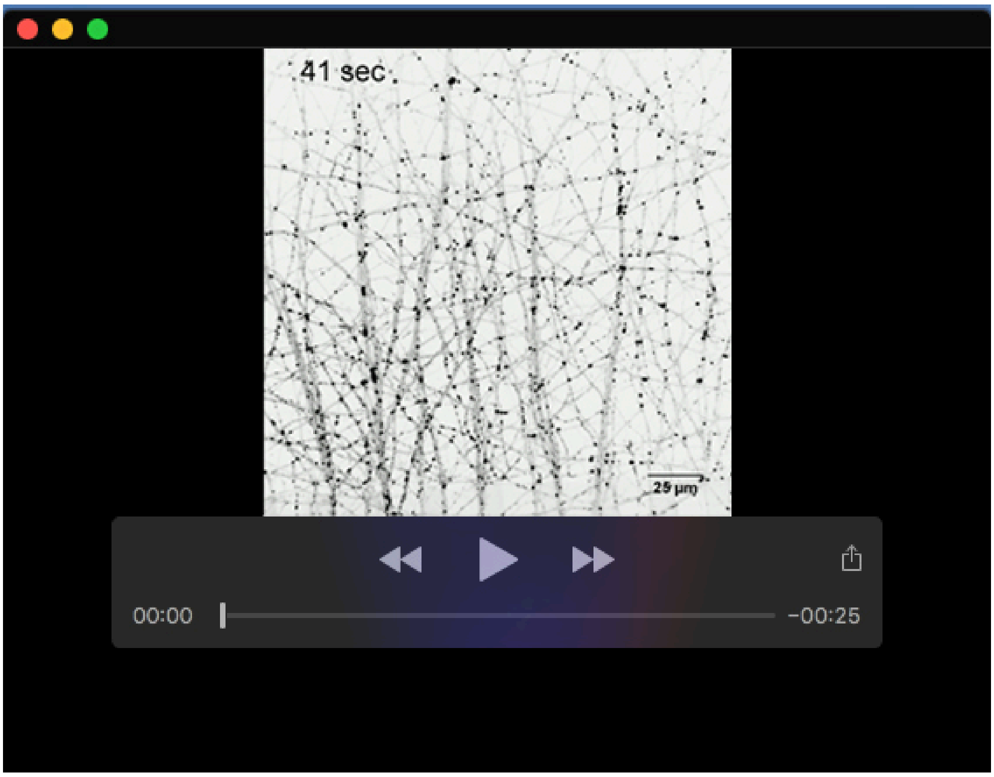


**Movie 2.** Corresponding to Fig. 4C. Movement of lysotracker-positive vesicles in the axonal compartment of WT neurons grown in 2-compartment MFCs imaged at 1 frame per second. Scale bar = 25 µm.

Journal of Cell Science • Supplementary information

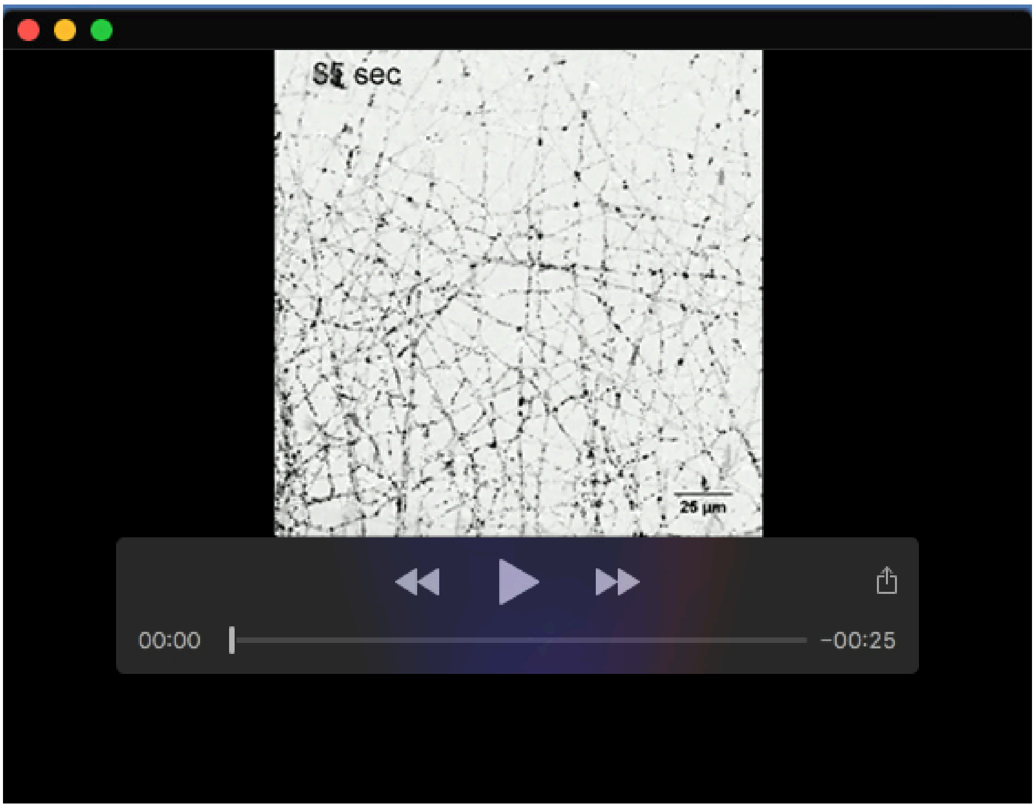


**Movie 3.** Corresponding to Fig. 4D. Movement of lysotracker-positive vesicles in the axonal compartment of TTL KO neurons grown in 2-compartment MFCs imaged at 1 frame per second. Scale bar = 25 µm.

Journal of Cell Science • Supplementary information

## Table S1. Crucial reagents and resources Primary antibodies

*Confocal and STED imaging*

| Target | Host | Supplier | Product number | Dilution |
|---|---|---|---|---|
| AnkyrinG | Mouse | Millipore | MABN466 | 1:500 |
| TRIM46 | Rabbit | Synaptic Systems | 377 003 | 1:500 |
| Beta-4-spectrin | Mouse | Neuromab | 75-377 | 1:500 |
| MAP2 | Mouse | Sigma | M4403 | 1:500 |
| MAP2 | Rabbit | Abcam | ab32454 | 1:500 |
| Detyr-α-tubulin | Rabbit | Abcam | ab48389 | 1:500 |
| Tubulin Tyrosine Ligase | Mouse | Proteintech | 66076-1-Ig | 1:500 |

*Western Blot – Fig. 1*

| | | | | |
|---|---|---|---|---|
| α-tubulin (α3A1) | Mouse | *Self-made (described in Aillaud et al., 2017)* | n.a. | 1:10000 |
| Tyr-α-tubulin (YL1/2) | Rat | | n.a. | 1:10000 |
| Detyr-α-tubulin | Rabbit | | n.a. | 1:10000 |

*Western Blot – Fig. 2*

| | | | | |
|---|---|---|---|---|
| Ankyrin-G | Mouse | EMD Millipore | MABN466 | 1:500 |
| TRIM46 | Rabbit | Proteintech | 30298-1-AP | 1:2000 |
| α-tubulin | Mouse | Sigma | T5168 | 1:2000 |

### Secondary antibodies

*Confocal Imaging*

| Target | Host | Conjugation | Supplier | Product number | Dilution |
|---|---|---|---|---|---|
| Mouse | Goat | Alexa Fluor 568 | Thermo Fisher | A-11031 | 1:500 |
| Rabbit | Goat | Alexa Fluor 488 | Thermo Fisher | A-21245 | 1:500 |
| Mouse | Goat | Alexa Fluor 594 | Thermo Fisher | A-11005 | 1:500 |

*STED Imaging*

| | | | | | |
|---|---|---|---|---|---|
| Mouse | Goat | Alexa Fluor 594 | Thermo Fisher | A-11005 | 1:500 |
| Mouse | Goat | Abberior Star Orange | Fisher Scientific | NC1933863 | 1:500 |

*Western Blot*

| | | | | | |
|---|---|---|---|---|---|
| Mouse | Goat | HRP | Dianova | 115-035-146 | 1:10 000 |
| Rabbit | Goat | HRP | Dianova | 111-035-144 | 1:10 000 |

### Other reagents

| Name | Supplier | Product number | Dilution |
|---|---|---|---|
| DAPI | Sigma | D9542 | 1:1000 |
| Lysotracker Green DND-26 | Thermo Fisher | L7526 | 1:10.000 |
| Lysotracker Red DND-99 | Thermo Fisher | L7528 | 1:20.000 |
| Magic Red Cathepsin-B Assay Kit | Biomol | ICT937 | 1:250 |
| Phalloidin-Atto647N | Sigma | 65906 | 1:50 |

### AAVs

| Name | Supplier | Viral titer | Dilution |
|---|---|---|---|
| AAV9 sh_5027 (control shRNA) produced from pAAV9-U6-shRNA_5027-EF1α-eGFP<br>shRNA sequence:<br>GGAATCTCATTCGATGCATACCTC<br>GAGGTATGCATCGAATGAGATTCC | Servier | 7.0^12 viral genomes/ml | 1:8000 |
| AAV9 sh_3616 (mouse TTL shRNA) produced from pAAV9-U6-shRNA_3616-EF1α-eGFP<br>shRNA sequence:<br>AAGTGCACGTGATCCAGAAATCTC<br>GAGATTTCTGGATCACGTGCACTT | Servier | 1.5^13 viral genomes/ml | 1:8000 |

## References

**Aillaud et al.** Vasohibins/SVBP are tubulin carboxypeptidases (TCPs) that regulate neuron differentiation. *Science*. 2017 Dec 15; **358** (6369):1448-1453.

Journal of Cell Science • Supplementary information
