# Supplementary material for: Efficient axonal transport of endolysosomes relies on the balanced ratio of microtubule tyrosination and detyrosination
Source: J Cell Sci. 2024 Apr 30;137(8):jcs261737. doi: 10.1242/jcs.261737 (PMC11112122; doi:10.1242/jcs.261737)
Supplement: Supplementary file 1 [file joces-137-261737_review_history.pdf]

Efficient axonal transport of endolysosomes relies on the balanced ratio of microtubule tyrosination and detyrosination

Anja Konietzny, Yuhao Han, Yannes Popp, Bas van Bommel, Aditi Sharma, Philippe Delagrange, Nicolas Arbez, Marie-Jo Moutin, Leticia Peris and Marina Mikhaylova
DOI: 10.1242/jcs.261737

Editor: Giampietro Schiavo

Review timeline

Original submission:	18 October 2023
Editorial decision:	24 November 2023
First revision received:	11 March 2024
Accepted:	14 March 2024

Original submission

First decision letter

MS ID#: JOCES/2023/261737

MS TITLE: Efficient axonal trafficking of endolysosomes depends on the balanced ratio of microtubule tyrosination and detyrosination

AUTHORS: Anja Konietzny, Leticia Peris, Yuhao Han, Yannes Popp, Bas van Bommel, Aditi Sharma, Philippe Delagrange, Nicolas Arbez, Marie-Jo Moutin, and Marina Mikhaylova
ARTICLE TYPE: Short Report

We have now reached a decision on the above manuscript.

To see the reviewers' reports and a copy of this decision letter, please go to: https://submit-jcs.biologists.org and click on the 'Manuscripts with Decisions' queue in the Author Area. (Corresponding author only has access to reviews.)

As you will see, the reviewers gave favourable reports but raised some critical points that will require amendments to your manuscript. I hope that you will be able to carry these out because I would like to be able to accept your paper, depending on further comments from reviewers.

Please ensure that you clearly highlight all changes made in the revised manuscript. Please avoid using 'Tracked changes' in Word files as these are lost in PDF conversion.

I should be grateful if you would also provide a point-by-point response detailing how you have dealt with the points raised by the reviewers in the 'Response to Reviewers' box. Please attend to all of the reviewers' comments. If you do not agree with any of their criticisms or suggestions please explain clearly why this is so.

Reviewer 1

Advance summary and potential significance to field

© 2024. Published by The Company of Biologists under the terms of the Creative Commons Attribution License (https://creativecommons.org/licenses/by/4.0/).

The manuscript by Konietzny et al titled “Efficient axonal trafficking of endolysosomes depends on the balanced ratio of microtubule tyrosination and detyrosination” reports that balance of microtubule tyrosination/detyrosination affects endolysosomes trafficking in the axons.

Tyrosinated microtubules are assembled from gene synthesized alpha-tubulin, while detyrosinated microtubules lack tyrosine on the C-terminal tail of the alpha-tubulin. Detyrosination of microtubules is catalyzed by Vasohibin1/2-small vasohibin binding protein (VASH-SVBP) complex. This modification is reversible and catalyzed by tubulin-tyrosine ligase (TTL).

In this manuscript, the authors show that axons of SVBP KO (increased tyrosination) and TTL KO (increased detyrosination) neurons affects the length of axon initial segment without any influence on the membrane-associated periodic cytoskeleton. The authors further show that changes in tyrosination/detyrosination ratio affects the transport of endolysosomes. Overall, the manuscript is well-written and the science it presents is interesting to not only microtubule community but also to the cell biology field. However, in this reviewer’s opinion, the data representation and analysis in the current form of the manuscript is a bit misleading and should be addressed to avoid confusion. Also, some experiments lack important technical details to properly assess the quality of the data and should be provided wherever appropriate. These minor issues should be fixed in the revision.

Comments for the author

Specific points:
1. The authors report that changing tubulin tyrosination/detyrosination ratio does not affect the periodic distribution of F-actin in the axon initial segment (Fig.1D). This experiment is not properly described. For example, there is no description about the staining or analysis in the materials section. Although there is no difference reported between the control and experimental condition, it is still important to statistically quantify these observations. How many axons were imaged for this analysis? How is the cut off (190 nm) shown in the Fig.1D intensity profile determined? In this figure, the cut off does not always correspond to the peaks of the intensity profiles. The figure panel shown here suggests that this imaging was performed in the axon initial segment (as labelled by authors in the figure panel). It is not mentioned how they identified the axon initial segment.
Were the neurons additionally labelled with AnkG or TRIM46?

2. The authors show that changes in tyrosination/detyrosination ratio in the axons affects the motility of lysotracker-positive organelles (Fig.3). This is one of the main messages of the manuscript and the authors should strengthen these data to make it fool-proof. In the current form, this manuscript lacks to provide important information pertaining to these experiments.

2a. In figure 3, the authors do not show representative kymographs for each condition, making it difficult to visually assess the motility behavior of lysosomes in the respective experimental conditions. Also, the authors should provide the corresponding movies for each condition.

2b. The transport experiments shown in Fig.3 uses lysotracker, that labels lysosomes. These lysotracker-positive organelles move predominantly in the retrograde direction, as shown in the literature and also evident in the supplementary example movie shown by the authors. However, according to the authors’ analysis the avg. run length of these organelles is very similar in anterograde and retrograde directions (Fig.3J compared with Fig.3L). This raises the question if authors’ definition of ‘run’ (consecutive movement for 5 seconds) informs on the actual physiological behavior of lysosomes. The authors should rethink about this part and maybe consider to also show total run length and duration in anterograde/retrograde directions for comparisons.

2c. The authors show ‘percent stationary tracks’ in Fig.3F. From their explanation of how they defined a ‘track’ (total tracing of a given lysosome), and the Y-axis label, it is understood that this is the percentage of immobile lysosomes in a given axon. In this case, each dot shown in Fig.3F should correspond to one axon value. However, the numbers provided for the number of axons analyzed do not appear to match with this idea. Could the authors clearly explain how these values (Percent stationary tracks and Avg. pause duration) are calculated and what each dot in the shown scatter plots represent?

© 2024. Published by The Company of Biologists under the terms of the Creative Commons Attribution License (https://creativecommons.org/licenses/by/4.0/).

2d. Throughout the manuscript, the authors show scatter plots showing the spread of the measurements, which is appreciated by this reviewer. However, it is not clear why the authors chose to show the horizontal line for mean for such a big spread and not median?

2e. For multiple comparisons, the authors mostly used Kruskall-Wallis test that compares the medians of the distributions, which is recommended given the inherent spread of the measurements. However, it is confusing why the authors chose to use ANOVA test for Fig. 3E.

3. Beta4-spectrin arrangement has been shown to be linked with AnkG localization. The authors should discuss how in their experiments changing tyrosination/detyrosination ratio affects AnkG localization but not beta4-spectrin.

4. The authors use lysotracker dye that labels mainly acidic lysosomes. It is confusing why the authors start referring to these lysotracker-positive organelles as endolysosomes. What experiments confirmed that these are endolysosomes?

5. The authors confirms that SVBP KO increases tyrosinated tubulin and TTL KO increases detyrosinated tubulin (Figs.1B and 1C). In the figure legend, they mention that the statistical significance was tested using Kruskall-Wallis test with Dunn's multiple comparison test. Is it simply a copy-paste clerical error?

6. It has been reported earlier that lysosomes transport is hindered on detyrosinated microtubules (Mohan et al 2019). The authors should discuss a bit more about these results in the context of their study.

7. The authors describe about mice genotyping in the materials and methods, but no associated figure is shown in the manuscript. The authors should consider to show the genotyping figure so the readers don't have to refer to other published papers.

<u>Reviewer 2</u>

Advance summary and potential significance to field

The manuscript by Konietzny A. et al. entitled "Efficient axonal trafficking of endolysosomes depends on the balanced ratio of microtubule tyrosination and detyrosination" describes the influence of decreased or increased levels of tubulin detyrosination on the length of the Axon Initial Segment (AIS) and lysosome trafficking. The authors find that abnormally high or abnormally low levels of detyrosination results in shorter AIS and decreased lysosome trafficking. Both observations are novel and potentially interesting, however the mechanistic link between them is rather unclear.

Comments for the author

Suggestions for the authors:
1. The authors present the evidence that changes in the detyrosination levels, regardless if it is an increase or decrease, lead to shorter AIS and reduced lysosome trafficking. Do they claim that the shorter AIS is the results of the lysosome trafficking defects? Or, are these two observations independent? At the moment this is unclear. If the claim is that reduced lysosome traffic leads to shorter AIS, it will need to be substantiated experimentally. If there is no link between the two phenotypes then their study is purely descriptive.
2. According to the quantification, the F-actin staining intensity in the TTL KO neurons appears to be similar to control. However, the image provided in figure 1F does not seem to reflect these quantifications as overall actin levels seem be strongly reduced and the size of the actin patches are increased. Can the authors comment on these discrepancies? Is it possible that the Membrane Associated Periodic Skeleton is after all affected in the TTL KO cells?
3. The conclusion that changes in the levels of detyrosination lead to the formation of the shorter AIS are based on the IF staining. One possible explanation for the shorter AIS is simply that the expression levels of the markers (TRIM46 and AnkG) are affected. This applies at least to the

© 2024. Published by The Company of Biologists under the terms of the Creative Commons Attribution License (https://creativecommons.org/licenses/by/4.0/).

SVBP neurons, where TRIM46 staining appears to be strongly reduced as compared to controls. Could the authors asses the expression levels in different conditions of at least TRIM46 using immunoblots?

Reviewer 3

Advance summary and potential significance to field

Peer review for JCS

The manuscript by Konietzny and colleagues presents and investigation on the disruption of tubulin tyrosination on axonal transport using two knock out mouse lines to disrupt the balance of tubulin tyrosination. TTL-KOs are shown to have almost exclusively detyrosinated tubulin whilst SVBP-KOs have almost mainly tyrosinated tubulin. At the level of the neuronal cell biology investigated, this produces very subtle effects. Firstly as previously reported, there is a multiple axon phenotype in TTL KO neurons but not SVBP-KOs. Secondly, there is a subtle shortening of the AIS in both KO populations. Finally, another subtle commonality in the slight disruption of endolysosomal trafficking in both KO neuron types.
Trafficking phenotypes are further verified by knockdown of TTL.

The manuscript is very well written and the data presented with exceptional clarity; I would really like to commend the authors for the effort put into its presentation because it made for a very enjoyable reading experience. More importantly, it is rare to encounter work in the study of molecular and cellular neurobiology that embraces subtle phenotypes rather than rigid molecular determinism. Consequently, I think the manuscript offers an important contribution to the discussion around microtubule modifications and neuronal cell biology. It also moves us closer to understanding the highly robust nature of the emergent properties of the neuronal cytoskeleton; it is fascinating that despite such radical changes to the surface profile of microtubules, the inherent polarisation and structure of neurons is robust. To me it is even more surprising that the transport and AIS are relatively unperturbed, when the multiple axon phenotype is so strong in the TTL KO mice.

Comments for the author

I did have some comments on areas where the manuscript could be stronger. Firstly I noticed that there was no discussion of the phenotype of the KO mice. I assume this means there is none, but an explicit reference in the text would be beneficial, particularly with reference to the age mice have been observed to be healthy so far. This is related to the current absence of any characterisation of neuronal function in the KO neurons. Although AIS and trafficking are studied in detail, there is no characterisation of the activity of the neurons; given that action potential propagation is tightly linked to the AIS it would be beneficial to know if the function of neurons is disrupted or still within the normal range.
Finally, there is no indication of whether the profiles of other tubulin PTMs in the KO mice remain unchanged e.g. acetylation, glycylation etc. These are important controls in order to understand if neurons are responding to the changed tyrosination state by modulating other PTM pathways to compensate.

First revision

Author response to reviewers' comments

Since formatting is lost in the text pasted below, for easier reading please see "Point-by-point reply to Reviewers" attached as a Supplementary PDF file

Point-by-point reply to Reviewers

© 2024. Published by The Company of Biologists under the terms of the Creative Commons Attribution License (https://creativecommons.org/licenses/by/4.0/).

We would like to thank all three Reviewers and the JCS editorial office for taking the time to provide us with very valuable feedback, which helped us to improve the manuscript. In light of the implemented changes, new data and extended discussion, the manuscript now has been reformatted from a Short Report into a full Article. All changes in the revised version are marked in blue.

Reviewer 1

Advance summary and potential significance to field The manuscript by Konietzny et al titled "Efficient axonal trafficking of endolysosomes depends on the balanced ratio of microtubule tyrosination and detyrosination" reports that balance of microtubule tyrosination/detyrosination affects endolysosomes trafficking in the axons.

Tyrosinated microtubules are assembled from gene synthesized alpha-tubulin, while detyrosinated microtubules lack tyrosine on the C-terminal tail of the alpha-tubulin. Detyrosination of microtubules is catalyzed by vasohibin1/2-small vasohibin binding protein (VASH-SVBP) complex. This modification is reversible and catalyzed by tubulin-tyrosine ligase (TTL).

In this manuscript, the authors show that axons of SVBP KO (increased tyrosination) and TTL KO (increased detyrosination) neurons affects the length of axon initial segment without any influence on the membrane-associated periodic cytoskeleton. The authors further show that changes in tyrosination/detyrosination ratio affects the transport of endolysosomes. Overall, the manuscript is well-written and the science it presents is interesting to not only microtubule community but also to the cell biology field. However, in this reviewer's opinion, the data representation and analysis in the current form of the manuscript is a bit misleading and should be addressed to avoid confusion. Also, some experiments lack important technical details to properly assess the quality of the data and should be provided wherever appropriate. These minor issues should be fixed in the revision.

Reviewer 1 Comments for the author

Specific points:
1. The authors report that changing tubulin tyrosination/detyrosination ratio does not affect the periodic distribution of F-actin in the axon initial segment (Fig.1D). This experiment is not properly described. For example, there is no description about the staining or analysis in the materials section. Although there is no difference reported between the control and experimental condition, it is still important to statistically quantify these observations. How many axons were imaged for this analysis? How is the cut off (190 nm) shown in the Fig.1D intensity profile determined? In this figure, the cut off does not always correspond to the peaks of the intensity profiles. The figure panel shown here suggests that this imaging was performed in the axon initial segment (as labelled by authors in the figure panel). It is not mentioned how they identified the axon initial segment. Were the neurons additionally labelled with AnkG or TRIM46?
Reply: We agree with the reviewer and thank them for pointing out this issue. We included a detailed description of the staining and imaging procedure in the materials section, and performed additional characterization of the F-actin structures present in the AIS. We identified the AIS for STED imaging by an accumulation of the AIS marker beta-4-spectrin and have now included a corresponding confocal image for clarification.

The mentioned 190 nm spacing of the submembrane periodic lattice has been independently reported in the literature by several research groups (Xu et al. 2013, Leterrier et al. 2015), and is determined by the physical size of the alpha-2- and beta-4-spectrin molecules that are stacked in between the F-actin rings. We had originally included the 190 nm vertical lines in the figure as a visual aid and to highlight that the submembrane lattice in the AIS of knockout neurons is formed normally.

We have now replaced this figure and instead quantified the degree of periodicity by performing an autocorrelation analysis on the phalloidin signal, and show that it exhibits a distribution with ~ 190 nm intervals in all genotypes, as expected (new Fig. 1F).

References:

© 2024. Published by The Company of Biologists under the terms of the Creative Commons Attribution License (https://creativecommons.org/licenses/by/4.0/).

Xu et al. (2013) Actin, Spectrin, and Associated Proteins Form a Periodic Cytoskeletal Structure in Axons. Science 339, 452-456.

Leterrier et al. (2015) Nanoscale Architecture of the Axon Initial Segment Reveals an Organized and Robust Scaffold. Cell Rep. 2015 Dec 29;13(12):2781-93.

2. The authors show that changes in tyrosination/detyrosination ratio in the axons affects the motility of lysotracker-positive organelles (Fig.3). This is one of the main messages of the manuscript and the authors should strengthen these data to make it fool-proof. In the current form, this manuscript lacks to provide important information pertaining to these experiments.

2a. In figure 3, the authors do not show representative kymographs for each condition, making it difficult to visually assess the motility behavior of lysosomes in the respective experimental conditions. Also, the authors should provide the corresponding movies for each condition.
Reply: To strengthen the main conclusion of the manuscript, as suggested by this reviewer we have included representative kymographs from each genotype in the new Figure 4, and added movies of the field of view from which the kymographs were taken in the supplement (Supplementary Movies 1-3).

2b. The transport experiments shown in Fig.3 uses lysotracker, that labels lysosomes. These lysotracker-positive organelles move predominantly in the retrograde direction, as shown in the literature and also evident in the supplementary example movie shown by the authors. However, according to the authors' analysis the avg. run length of these organelles is very similar in anterograde and retrograde directions (Fig.3J compared with Fig.3L). This raises the question if authors' definition of 'run' (consecutive movement for 5 seconds) informs on the actual physiological behavior of lysosomes. The authors should rethink about this part and maybe consider to also show total run length and duration in anterograde/retrograde directions for comparisons.
Reply: The reviewer raises a good point here, and we acknowledge that we didn't word our results clearly enough in the first version of the manuscript. While we did observe a trend towards retrograde movement in the WT (about 60% of mobile lysotracker-positive organelles moved retrogradely, and 40% anterogradely, in old Figure 3H), this effect was not as pronounced as what has been reported by Lie et al. (2021), who showed that acidified organelles positive for the lysosome-marker LAMP1 moved almost exclusively in the retrograde direction. We have now included a more detailed discussion that compares our results with those of Lie et al. and provided possible explanations.

It is also true that looking at our example movies, one gets the impression of predominant retrograde movement of lysotracker-positive organelles. This is due to the fact that, judging by eye, large and bright organelles tend to move retrogradely. However, our kymograph analysis also included dimly positive organelles (see new Fig. 4B-D), which tended to move bidirectionally. It is likely that strongly labelled and weakly labelled organelles represent different organelle populations or subtypes, however it was not the aim of this study to differentiate between minute organelle identities, but to rather get a general overview of MT-based transport processes in the axon, and how they are affected by changes in MT Tyr/deTyr state.
Finally, to show our results more clearly, we have replaced the old figure panel 3H with a different graph that shows the "average net displacement" of lysotracker-positive organelles in µm, again showing that overall net displacement of lysotracker-positive organelles is slightly skewed in the retrograde direction in WT (new Fig. 4H).

2c. The authors show 'percent stationary tracks' in Fig.3F. From their explanation of how they defined a 'track' (total tracing of a given lysosome), and the Y-axis label, it is understood that this is the percentage of immobile lysosomes in a given axon. In this case, each dot shown in Fig.3F should correspond to one axon value. However, the numbers provided for the number of axons analyzed do not appear to match with this idea. Could the authors clearly explain how these values (Percent stationary tracks and Avg. pause duration) are calculated and what each dot in the shown scatter plots represent?
Reply: The Reviewer is absolutely correct in pointing this out, there has been a mistake in the figure legend regarding the n-numbers. We have fixed the mistake, and corrected the description in

© 2024. Published by The Company of Biologists under the terms of the Creative Commons Attribution License (https://creativecommons.org/licenses/by/4.0/).

new Fig. 4 (n=58, 164, 114 axons from SVBP (3 independent experiments), WT (6 independent experiments) and TTL KO (5 independent experiments), respectively).

2d. Throughout the manuscript, the authors show scatter plots showing the spread of the measurements, which is appreciated by this reviewer. However, it is not clear why the authors chose to show the horizontal line for mean for such a big spread and not median?
Reply: The reviewer is right, displaying the median is more suitable for this type of data. We have now updated all graphs accordingly.

2e. For multiple comparisons, the authors mostly used Kruskall-Wallis test that compares the medians of the distributions, which is recommended given the inherent spread of the measurements. However, it is confusing why the authors chose to use ANOVA test for Fig. 3E.
Reply: This was indeed an error in the figure legend – it should have referred to old Fig. 3H instead of old Fig. 3E. This figure panel has now been replaced with a different graph (new Fig. 4H), now showing “average net displacement” in μm, instead of “percent displacement”. All graphs in this new Fig. 4 have been analyzed using Kruskal-Wallis test.

3. Beta4-spectrin arrangement has been shown to be linked with AnkG localization. The authors should discuss how in their experiments changing tyrosination/detyrosination ratio affects AnkG localization but not beta4-spectrin.
Reply: In this study, we did not analyze beta-4-spectrin and only used this labeling to identify the AIS in experiments where we analyzed the F-actin structures (new Fig. 1D-H).

4. The authors use lysotracker dye that labels mainly acidic lysosomes. It is confusing why the authors start referring to these lysotracker-positive organelles as endolysosomes. What experiments confirmed that these are endolysosomes?
Reply: As the reviewer points out, Lysotracker labels acidic organelles, which in non-neuronal cells are mostly represented by lysosomes. In neurons, many transitory states in the maturation of lysosomes have been observed, and the identity and nomenclature of such organelles is still under debate. To make the picture even more complex, there is a discrepancy in the literature regarding the existence of mature lysosomes in distal axons. Some papers claim that they do exist, while others suggest they do not (Farfel-Becker et al., 2019; Lie et al., 2021). This discrepancy may be attributed to the different ages of neuronal cultures and culturing methods used in these publications. Since this manuscript focuses on the role of MT tyrosination and detyrosination in axonal transport processes, for simplicity’s sake we decided to use the term ‘endolysosomes’ to refer to acidic organelles positive for Lysotracker. We have now included a more detailed explanation of this situation in the main text.

References:
Lie et al. (2021) Post-Golgi carriers, not lysosomes, confer lysosomal properties to pre-degradative organelles in normal and dystrophic axons. Cell Rep. 2021 Apr 27;35(4):109034.
Farfel-Becker et al. (2019) Neuronal Soma-Derived Degradative Lysosomes Are Continuously Delivered to Distal Axons to Maintain Local Degradation Capacity. Cell Rep. 2019 Jul 2;28(1):51-64.e4.

5. The authors confirms that SVBP KO increases tyrosinated tubulin and TTL KO increases detyrosinated tubulin (Figs.1B and 1C). In the figure legend, they mention that the statistical significance was tested using Kruskall-Wallis test with Dunn’s multiple comparison test. Is it simply a copy-paste clerical error?
Reply: Indeed, this was an error in the figure legend carried over from an earlier version, which we have now corrected. The performed test was indeed a Mann Whitney test. It is corrected in the revised version.

6. It has been reported earlier that lysosomes transport is hindered on detyrosinated microtubules (Mohan et al 2019). The authors should discuss a bit more about these results in the context of their study.
Reply: Discussion on the above-mentioned point is now incorporated in the manuscript.

© 2024. Published by The Company of Biologists under the terms of the Creative Commons Attribution License (https://creativecommons.org/licenses/by/4.0/).

7. The authors describe about mice genotyping in the materials and methods, but no associated figure is shown in the manuscript. The authors should consider to show the genotyping figure so the readers don't have to refer to other published papers.
Reply: We have included a more detailed explanation of the genotyping strategy (Fig. S1) and images of gels in the supplement (Figure S5).

*****

Reviewer 2

The manuscript by Konietzny A. et al. entitled "Efficient axonal trafficking of endolysosomes depends on the balanced ratio of microtubule tyrosination and detyrosination" describes the influence of decreased or increased levels of tubulin detyrosination on the length of the Axon Initial Segment (AIS) and lysosome trafficking. The authors find that abnormally high or abnormally low levels of detyrosination results in shorter AIS and decreased lysosome trafficking. Both observations are novel and potentially interesting, however the mechanistic link between them is rather unclear.

Reviewer 2 Comments for the author

Suggestions for the authors:

1.The authors present the evidence that changes in the detyrosination levels, regardless if it is an increase or decrease, lead to shorter AIS and reduced lysosome trafficking. Do they claim that the shorter AIS is the results of the lysosome trafficking defects? Or, are these two observations independent? At the moment this is unclear. If the claim is that reduced lysosome traffic leads to shorter AIS, it will need to be substantiated experimentally. If there is no link between the two phenotypes then their study is purely descriptive.
Reply: In this study we focused on describing the role of MT tyrosination and detyrosination on axonal transport. The AIS is an important structure that separates the axon from the somato-dendritic compartment. It serves as a diffusion barrier, participates in axonal cargo sorting, and due to its role in ion channel clustering, the AIS structure is important for generation of action potentials. The AIS has a unique MT arrangement (MT fascicles) and specialized membrane organization. Along the axon, most microtubules have the minus end pointing towards the periphery and MT detyrosination is highly abundant there. Since MT detyrosination guides kinesin-1 and several other motor proteins (Lavrsen et al. 2023), it is possible that organelle trafficking in axons will be affected when Tyr/deTyr levels become unbalanced. To test this hypothesis, we decided to investigate endolysosomal trafficking, as those organelles play several important functions in axons and they are associated with kinesin-1 motor. Our findings on the shortening of the AIS and reduced lysosomal trafficking in the TTL and SVBP KO neurons are two independent observations. They are not necessary causative, which we will state more clearly in the manuscript. In our opinion it is also important to provide descriptive data on structural and functional features of axons upon change of one of the most prominent MT PTMs.

Reference:
Lavrsen et al. (2023) Microtubule detyrosination drives symmetry breaking to polarize cells for directed cell migration. Proc Natl Acad Sci U S A. 2023 May 30;120(22):e2300322120. doi: 10.1073/pnas.2300322120.

2.According to the quantification, the F-actin staining intensity in the TTL KO neurons appears to be similar to control. However, the image provided in figure 1F does not seem to reflect these quantifications as overall actin levels seem be strongly reduced and the size of the actin patches are increased. Can the authors comment on these discrepancies? Is it possible that the Membrane Associated Periodic Skeleton is after all affected in the TTL KO cells?
Reply: We have now included a more detailed analysis of the F-actin structures in the AIS observed by STED imaging, which do not show any significant changes between the genotypes in the new Figure 1. (See also comment 1 by Reviewer 1). We agree with the reviewer that our examples might have been misleading. We have replaced them by more representative images. Based on our analysis, we have no reason to assume that the F-actin cytoskeleton of the AIS is altered in a

© 2024. Published by The Company of Biologists under the terms of the Creative Commons Attribution License (https://creativecommons.org/licenses/by/4.0/).

significant way in either TTL KO or SVBP KO. It is also important to note that the levels of fluorescence intensity in the example STED images do not necessarily correlate with the amount of protein present.

3.The conclusion that changes in the levels of detyrosination lead to the formation of the shorter AIS are based on the IF staining. One possible explanation for the shorter AIS is simply that the expression levels of the markers (TRIM46 and AnkG) are affected. This applies at least to the SVBP neurons, where TRIM46 staining appears to be strongly reduced as compared to controls. Could the authors asses the expression levels in different conditions of at least TRIM46 using immunoblots?
Reply: Thank you for this suggestion. To test this, we now included additional experiments and Western blot analysis of TRIM46 and AnkG amount present in lysate from TTL KO and SVBP KO hippocampal cultures and we found no statistically significant difference in the amount of either protein compared to lysate from WT cultures (New Fig. 2A-B). However, there seemed to be a non-significant trend in the case of AnkG to lowered amount of protein in TTL KO, and increased amount of protein in SVBP KO.

*****
Reviewer 3

The manuscript by Konietzny and colleagues presents and investigation on the disruption of tubulin tyrosination on axonal transport using two knock out mouse lines to disrupt the balance of tubulin tyrosination. TTL-KOs are shown to have almost exclusively detyrosinated tubulin whilst SVBP-KOs have almost mainly tyrosinated tubulin. At the level of the neuronal cell biology investigated, this produces very subtle effects. Firstly as previously reported, there is a multiple axon phenotype in TTL KO neurons but not SVBP-KOs. Secondly, there is a subtle shortening of the AIS in both KO populations. Finally, another subtle commonality in the slight disruption of endolysosomal trafficking in both KO neuron types. Trafficking phenotypes are further verified by knockdown of TTL.

The manuscript is very well written and the data presented with exceptional clarity; I would really like to commend the authors for the effort put into its presentation because it made for a very enjoyable reading experience. More importantly, it is rare to encounter work in the study of molecular and cellular neurobiology that embraces subtle phenotypes rather than rigid molecular determinism. Consequently, I think the manuscript offers an important contribution to the discussion around microtubule modifications and neuronal cell biology. It also moves us closer to understanding the highly robust nature of the emergent properties of the neuronal cytoskeleton; it is fascinating that despite such radical changes to the surface profile of microtubules, the inherent polarisation and structure of neurons is robust. To me it is even more surprising that the transport and AIS are relatively unperturbed, when the multiple axon phenotype is so strong in the TTL KO mice.

Comments for the author

I did have some comments on areas where the manuscript could be stronger. Firstly, I noticed that there was no discussion of the phenotype of the KO mice. I assume this means there is none, but an explicit reference in the text would be beneficial, particularly with reference to the age mice have been observed to be healthy so far. This is related to the current absence of any characterisation of neuronal function in the KO neurons. Although AIS and trafficking are studied in detail, there is no characterisation of the activity of the neurons; given that action potential propagation is tightly linked to the AIS it would be beneficial to know if the function of neurons is disrupted or still within the normal range. Finally, there is no indication of whether the profiles of other tubulin PTMs in the KO mice remain unchanged e.g. acetylation, glycylation etc. These are important controls in order to understand if neurons are responding to the changed tyrosination state by modulating other PTM pathways to compensate.

Reply: We would like to thank this reviewer for the positive evaluation of our work and for acknowledging the importance of reporting subtle effects, because in our opinion they are equally important to report. Following the reviewer’s suggestions, we now changed the format of the manuscript from a short report to a full article which allows a more elaborate discussion.

© 2024. Published by The Company of Biologists under the terms of the Creative Commons Attribution License (https://creativecommons.org/licenses/by/4.0/).

The reviewer further pointed out an intriguing observation regarding the profile of other PTMs in the KO mice, suggesting a compensatory mechanism to regulate changes in tubulin tyrosination. Tubulin glycylation was not examined, as it is known to modify microtubules specifically in cilia and flagella and appears to be absent in the neuronal microtubular network (Gadadhar et al., 2021). Instead, we investigated the interplay between acetylation and detyrosination and found a clear correlation between the two PTMs: tubulin detyrosination leads to tubulin acetylation (Martinez-Hernandez et al., 2022). Given that both PTMs are linked to increased microtubule stabilization and recognized by the same family of molecular motors (Sanyal et al., 2023), it seems that rather than acting as a compensatory mechanism, the additional increase of tubulin acetylation might exacerbate the phenotype by promoting further microtubule stabilization and transport defects.

References:
Gadadhar et al. (2021) Tubulin glycylation controls axonemal dynein activity, flagellar beat, and male fertility. Science. 2021 Jan 8;371(6525):pii: eabd4914. doi: 10.1126/science.abd4914.
Martínez-Hernández et al. (2022) Crosstalk between acetylation and the tyrosination/detyrosination cycle of α-tubulin in Alzheimer's disease. Front Cell Dev Biol. 2022 Aug 26;10:926914. doi: 10.3389/fcell.2022.926914.
Sanyal et al. (2021) The detyrosination/re-tyrosination cycle of tubulin and its role and dysfunction in neurons and cardiomyocytes. Semin Cell Dev Biol. 2023 Mar 15;137:46-62. doi: 10.1016/j.semcdb.2021.12.006.

Second decision letter

MS ID#: JOCES/2023/261737

MS TITLE: Efficient axonal transport of endolysosomes relies on the balanced ratio of microtubule tyrosination and detyrosination

AUTHORS: Anja Konietzny, Yuhao Han, Yannes Popp, Bas van Bommel, Aditi Sharma, Philippe Delagrange, Nicolas Arbez, Marie-Jo Moutin, Leticia Peris, and Marina Mikhaylova
ARTICLE TYPE: Research Article

I am happy to tell you that your manuscript has been accepted for publication in Journal of Cell Science, pending standard ethics checks.

© 2024. Published by The Company of Biologists under the terms of the Creative Commons Attribution License (https://creativecommons.org/licenses/by/4.0/).
